# An Insight into microRNA156 Role in Salinity Stress Responses of Alfalfa

**DOI:** 10.3389/fpls.2017.00356

**Published:** 2017-03-14

**Authors:** Muhammad Arshad, Margaret Y. Gruber, Ken Wall, Abdelali Hannoufa

**Affiliations:** ^1^Agriculture and Agri-Food Canada, LondonON, Canada; ^2^Agriculture and Agri-Food Canada, SaskatoonSK, Canada; ^3^Agriculture and Agri-Food Canada, Swift CurrentSK, Canada

**Keywords:** salt stress, miR156, alfalfa, biomass, ions

## Abstract

Salinity is one of the major abiotic stresses affecting alfalfa productivity. Developing salinity tolerant alfalfa genotypes could contribute to sustainable crop production. The functions of microRNA156 (miR156) have been investigated in several plant species, but so far, no studies have been published that explore the role of miR156 in alfalfa response to salinity stress. In this work, we studied the role of miR156 in modulating commercially important traits of alfalfa under salinity stress. Our results revealed that overexpression of miR156 increased biomass, number of branches and time to complete growth stages, while it reduced plant height under control and salinity stress conditions. We observed a miR156-related reduction in neutral detergent fiber under non-stress, and acid detergent fiber under mild salinity stress conditions. In addition, enhanced total Kjeldahl nitrogen content was recorded in miR156 overexpressing genotypes under severe salinity stress. Furthermore, alfalfa genotypes overexpressing miR156 exhibited an altered ion homeostasis under salinity conditions. Under severe salinity stress, miR156 downregulated *SPL* transcription factor family genes, modified expression of other important transcription factors, and downstream salt stress responsive genes. Taken together, our results reveal that miR156 plays a role in mediating physiological and transcriptional responses of alfalfa to salinity stress.

## Introduction

Salinity is a major threat to crop productivity and yield. Presently, almost half of the world’s irrigated land and about 20% of global cultivated land is affected by salinity (Paul and Lade, 2014). Ions such as Na^+^ present in the outside media enter into root cells establishing a large electrochemical gradient, which promotes passive influx of salt ions through channels and transporters located on the plasma membrane (Sun et al., 2009). This Na^+^ influx raises sodium concentration inside the cytosol triggering K^+^ efflux, which disturbs the K^+^/Na^+^ ratio and thereby intensifies salt stress conditions (Sun et al., 2009; Britto et al., 2010). Salinity also exerts osmotic and ionic stress in plants, which decreases plant root growth and leaf expansion. In the long term, Na^+^ and Cl^-^ accumulate in cells to toxic levels causing intrinsic injury leading to premature chlorosis or death of leaves (Munns, 2002).

Legume crops play a distinct role in ecological and agricultural aspects due to their ability to interact symbiotically with soil microorganisms to form nodules and fix atmospheric nitrogen (Wezel et al., 2014). Alfalfa (*Medicago sativa*) is an important legume crop that is not only used as forage for animal consumption but also to enhance soil fertility (Graham and Vance, 2003). Alfalfa cultivars carry large genetic diversity ranging from susceptible to tolerant to moderate salinity, but severe salt stress drastically reduces growth and productivity of alfalfa (Munns and Tester, 2008; Tuoxiong et al., 2010; Long et al., 2012; Steppuhn et al., 2012; Wang B.-P. et al., 2013). Salinity negatively affects plant growth, biomass production, forage quality, and nitrogen and antioxidant levels in plants (Parvaiz and Satyawati, 2008; Wang et al., 2009; Li et al., 2010).

MicroRNAs (miRNAs) are sequence-specific regulators of post transcriptional gene expression in eukaryotes. As non-protein coding genes, *miRNA*s are predominantly transcribed by RNA polymerase II, which requires Mediator with a conserved multi-subunit complex and the RNA splicing mechanism (Kim et al., 2011; Bielewicz et al., 2013). The transcription of *miRNA* genes results in production of a primary miRNA transcript containing a stem loop structure, which is further processed into an active mature miRNA in two steps. First, RNase III endoribonuclease removes the stem loop – the miRNA precursor from the primary miRNA transcript. Secondly, this miRNA precursor matures by forming a -5p/-3p miRNA duplex with two nucleotide 3′ overhangs. The mature miRNA targets genes by interacting with a complex containing an ARGONAUTE protein (Couzigou and Combier, 2016). In plants, miRNA regulates gene expression mainly by transcript cleavage or translational inhibition (Brodersen et al., 2008; Rogers and Chen, 2013; Reis et al., 2015). The miRNAs detect their targets by binding to (1) miRNA complementary sequences known as miRNA responsive elements, (2) 5′ untranslated regions or (3), 3′ untranslated regions (Rhoades et al., 2002; Jones-Rhoades and Bartel, 2004; Allen et al., 2005). Although plant miRNAs affect a range of genes that encode proteins with several functions, transcription factors remain their main targets (Hobert, 2004, 2008).

Plant microRNAs affect a variety of plant physiological traits such as root growth, apical dominance, plant biomass, flowering time, fruit and seed development, and environmental stress responses (Zhang and Wang, 2015). A study by (Wang M. et al., 2013) showed altered expression of five microRNAs (miR156, miR162, miR159, miR395, and miR396) in response to salt stress in cotton. MicroRNA156 (miR156) is conserved across plants and its role has widely been investigated in a variety of crops, model plants and tree species (Aung et al., 2015b; Wang and Wang, 2015). MiR156 delays flowering and increase root development in alfalfa, enhances biomass accumulation in switchgrass and leaf number in tomato as well as improves heat stress memory and abiotic stress tolerance in *Arabidopsis* (Fu et al., 2012; Cui et al., 2014; Stief et al., 2014; Aung et al., 2015a; Wang et al., 2015).

*SQUAMOSA PROMOTER BINDING PROTEIN-LIKE (SPL)* is a family of transcription factors regulated by microRNA156, and *SPLs* further regulate expression of downstream genes involved in specific traits (Aung et al., 2015b). Biological processes regulated by SPLs in plants include phase change from vegetative to reproductive, secondary metabolites synthesis and stress responses. MiR156-mediated downregulation of three *SPL* genes (*SPL2, SPL9*, and *SPL11*) enhanced plant response to heat stress in *Arabidopsis* (Stief et al., 2014). Similarly, miR156/SPL module positively affected root growth and secondary metabolite accumulation, leading to improvement of stress tolerance in *Arabidopsis* (Cui et al., 2014).

Other transcription factors including zinc finger proteins (ZFPs), WRKY, Apetala 2/Ethylene Response Factors (AP2/ERF) and basic region/leucine zipper motif (bZIP) have been described as important modulators of plant response to environmental stress (Singh et al., 2002; Ciftci-Yilmaz and Mittler, 2008; Jiang and Deyholos, 2009; Pandey and Somssich, 2009; Rushton et al., 2010; Mizoi et al., 2012; Shi et al., 2014; Hu et al., 2016). Overexpression of alfalfa AP2/ERF family genes (*MsERF8* and *MsERF11*) enhanced tolerance to salinity in tobacco and *Arabidopsis* (Chen et al., 2012a,b). Similarly, overexpression of *Glycine soja ZFP* and *WRKY20* improved drought and salt tolerance in alfalfa (Tang et al., 2013, 2014). Several studies showed involvement of bZIP transcription factor family genes in leaf senescence and modulating responses to various abiotic stresses including salinity and drought (Lee et al., 2006, 2010; Zou et al., 2008). Overexpression of stress-induced alfalfa bZIP (*MsZIP*) resulted in increased proline accumulation, soluble sugar content, relative water content and soluble protein content under drought and salt conditions in tobacco, which subsequently led to enhanced drought and salt stress tolerance (Li et al., 2013).

Abiotic stresses such as drought, salinity, and cold, regulate the expression of thousands of genes in plants at the transcriptional and the post transcriptional levels but the underlying molecular mechanism of salt tolerance in plants remains elusive (Zhu, 2002; Munns and Tester, 2008). Salinity induces expression of numerous genes to facilitate adaptive and defense responses in plants. The transcriptomes of two salt-tolerant alfalfa breeding populations were much more able to maintain their non-saline transcript levels and diversity under high saline conditions compared with a salt-sensitive population (Gruber et al., 2016). In terms of individual genes, salt-induced glycine rich plant proteins (GRPs) are characterized by their high content and repetitive sequences of glycine residues (Mangeon et al., 2010). Many GRPs are associated with the vascular system that pinpoints their role in stress responses (Ringli et al., 2001). Overexpression of alfalfa *GRP* increases sensitivity to ABA and abiotic stress in *Arabidopsis* (Long et al., 2013). A cytokinin receptor homolog (*MsHK1*) isolated from alfalfa root nodule showed induced expression under salt stress, indicating a potential role in salinity response (Coba de la Pena et al., 2008). The ability of plants to compartmentalize salt ions into vacuoles is considered a critical step toward maintenance of ion homeostasis inside the cell (Parida and Das, 2005). A plant tonoplast Na^+^/H^+^ antiporter, *NHX*, facilitates this crucial compartmentalisation step, and salt stress was shown to upregulate *NHX* expression in plants (Silva and Geros, 2009). *AtNHX1* overexpression mediated potassium transport and affected salinity response in tomato (Leidi et al., 2010). Transgenic alfalfa overexpressing the wheat *NHX2* exhibited improved salt tolerance (Zhang et al., 2015). Furthermore, overexpression of alfalfa *Rare Cold Inducible 2* (*RCI2*) enhanced tolerance to salt stress in *Arabidopsis* (Long et al., 2015).

In this study, we examined the role of miR156 in alfalfa salinity stress response. Our objective was to investigate the effects of miR156 in modulating the physiological and molecular mechanisms known to play a role in salt stress tolerance and forage nutrition. To our knowledge, this is the first study reporting a positive role for miR156 in salinity response of alfalfa.

## Materials and Methods

### Experimental Design

Plant growth experiments were conducted using hydroponic tanks with saline solutions at the Salt Laboratory of Agriculture and Agri-Food Canada in Swift Current, Saskatchewan as described in Steppuhn et al. (2012), but with the following variations. We tested transgenic alfalfa genotypes overexpressing microRNA156 (miR156OE) and plants transformed with an empty vector (EV) that were generated by Aung et al. (2015a). In addition to genotypes A8, A11, and A11a described in (Aung et al. (2015a), we also included an additional genotype (A4a) with relatively low miR156 expression in our study (Supplementary Figure 1A). MiR156OE and EV plants were grown starting from foam plugs of rooted cuttings in tanks that featured three cuttings per genotype and five genotypes (EV and 4 miR156OE) randomly distributed in each tank. The overall experimental design included four tanks for each of three electroconductivity (EC) salt levels (i.e., EC 1.4 dSm^-1^, 7.0 dSm^-1^, and 14 dSm^-1^). All 12 tanks were randomized for location in a greenhouse under an average of 16 h daylight and 20°C temperature conditions (Supplementary Figure 1B). EC 1.4 dSm^-1^, 7.0 dSm^-1^, and 14 dSm^-1^ were designated as control, mild and severe salinity stress, respectively.

### Physiological Data Collection

Physiological data were collected from four experiments (i.e., four re-growth harvests of forage material collected prior to blooming). For the first harvest, data were collected after 70 days of plant transplantation whereas for subsequent second, third, and fourth harvests, data were collected 30–35 days after the preceding harvest. Plant height was measured from the rooted cutting plug to the tip of the longest shoot. For root sample (4th harvest) collections, tanks were flooded to facilitate soil loosening. Alfalfa developmental stages (stage 0; early vegetative, stage 1;mid-vegitative, stage 2; late vegetative, stage 3; early bud, stage 4; late bud, stage 5; early flower) were determined from four harvests by examining the stems as described in Information Bulletin 217 (Fick and Mueller, 1989).

Leaf and root samples for RNA extraction, antioxidant and ion analysis were collected from the fourth harvest. Leaf RNA samples were collected from the tip of two shoots with no flower buds, three plants per genotype from each tank. Leaf samples for antioxidants were collected from two healthy shoots including tiny buds. RNA and antioxidant samples were frozen immediately in liquid nitrogen. Plant samples were first weighed to determine fresh weight or biomass. Dry weight was obtained by incubating samples in a forage drum drier at 40°C for 1–2 weeks. Dried samples from the fourth harvest were then ground for ions and nutrition analysis. Twenty centimeter-long root samples (measuring from the longest tip) were collected for RNA extraction from three plants per genotype per tank. Samples were then frozen immediately in liquid nitrogen.

### Determining Fiber and Nitrogen Content

To assess forage quality, we determined the fiber content in alfalfa leaf material collected from the fourth harvest. Neutral detergent fiber (NDF) levels were measured using an ANKOM200 fiber analyzer (Model 200; ANKOM; Fairport, NY, USA) according to the ANKOM Neutral Detergent Fiber in Feeds Filter Bag Technique (Ferreira and Mertens, 2007). For acid detergent fiber (ADF), we followed a protocol described in Goering and Van Soest (1970). For total Kjeldahl nitrogen (TKN) measurements, we used a H_2_SO_4_/Se/Na_2_SO_4_ digestion method as described in Varley (1966), and analysis was performed following a published method (Noel and Hambleton, 1976).

### Ion Analysis

Concentration of ions (Na^+^, Ca^2+^, Mg^2+^, and Zn^2+^) was measured from leaves of 1-month-old plants from the fourth harvest using HClO_4_/HNO_3_ digestion method (Jones, 1991) in the ICP-OES (Inductively Coupled Plasma – Optical Emission Spectroscopy) on a Fisher Scientific iCAP6300 Duo machine by following manufacturer’s instructions. Briefly, 1 g sample was taken into PTFE high pressure vessels, and 5 mL of concentrated nitric acid and 5 mL water were added for digestion in Milestone Ethos EZ microwave. The digest was diluted with ultra-pure water before running the samples along with blanks for each digestion cycle. For data analysis, the instrument was calibrated, and samples were analyzed. We performed method detection limit study by analyzing three digestion blanks and the average values were taken for detection limits.

### Antioxidant Measurements

Total antioxidant levels were determined from 1-month-old leaf samples (100 mg) using an antioxidant assay kit (Sigma–Aldrich, Oakville ON, Canada; catalog number CS0790) following the manufacturer’s instructions. Antioxidant reactions were performed in a 96-well plate by reading endpoint absorbance at 405 nm using a plate reader (BioTek, Synergy 2, Winooski, VT, USA). Concentration of antioxidants was calculated using a Trolox^TM^ standard curve (Supplementary Figure 2).

### Gene Expression Analysis

Leaf and root samples were ground using mortar and pestle into fine powder and 100 mg representative sample was used for RNA extraction using the PowerPlant RNA Isolation Kit (Mo Bio Laboratories Inc., San Diego, CA, USA). Extracted RNA was treated with TURBO DNase (Ambion, Austin, TX, USA). One microgram of total RNA was used for cDNA synthesize using an iScript cDNA synthesis kit (Bio-Rad Laboratories, Mississauga, ON, Canada). Gene amplification was performed in a C1000 Touch^TM^ Thermocycler Real-Time PCR System (Bio-Rad) using SsoFast SYBR Green Master Mix (Bio-Rad). QRT-PCR conditions were 1 cycle at 95°C for 30 s, then 40 cycles at 95°C for 5 s, 60°C for 15 s, followed by melting curve 65–95°C with 5 s/step, +0.5°C/cycle. QRT-PCR data was analyzed using a CFX-Manager (Bio-Rad) with a 2^-ΔCT^ method (Schmittgen and Livak, 2008). Suitable reference genes (*ACC1, Actin*) were selected (Supplementary Figure 3) and primers for reference and target genes were designed from *M. sativa* sequences (Gao et al., 2016) and are listed in Supplementary Table 1.

### Statistical Analysis

GraphPad Prism software was used to statistically analyze the data. The *t*-test or Analysis of Variance (ANOVA) were used to test the significance followed by Tukey’s HSD test for multiple comparisons. Statistical analyses were conducted on a sample size of 3–12 and *P*-value < 0.05.

## Results

### MiR156 Improves Biomass Accumulation in Alfalfa under Salinity Stress

**FIGURE 1:**
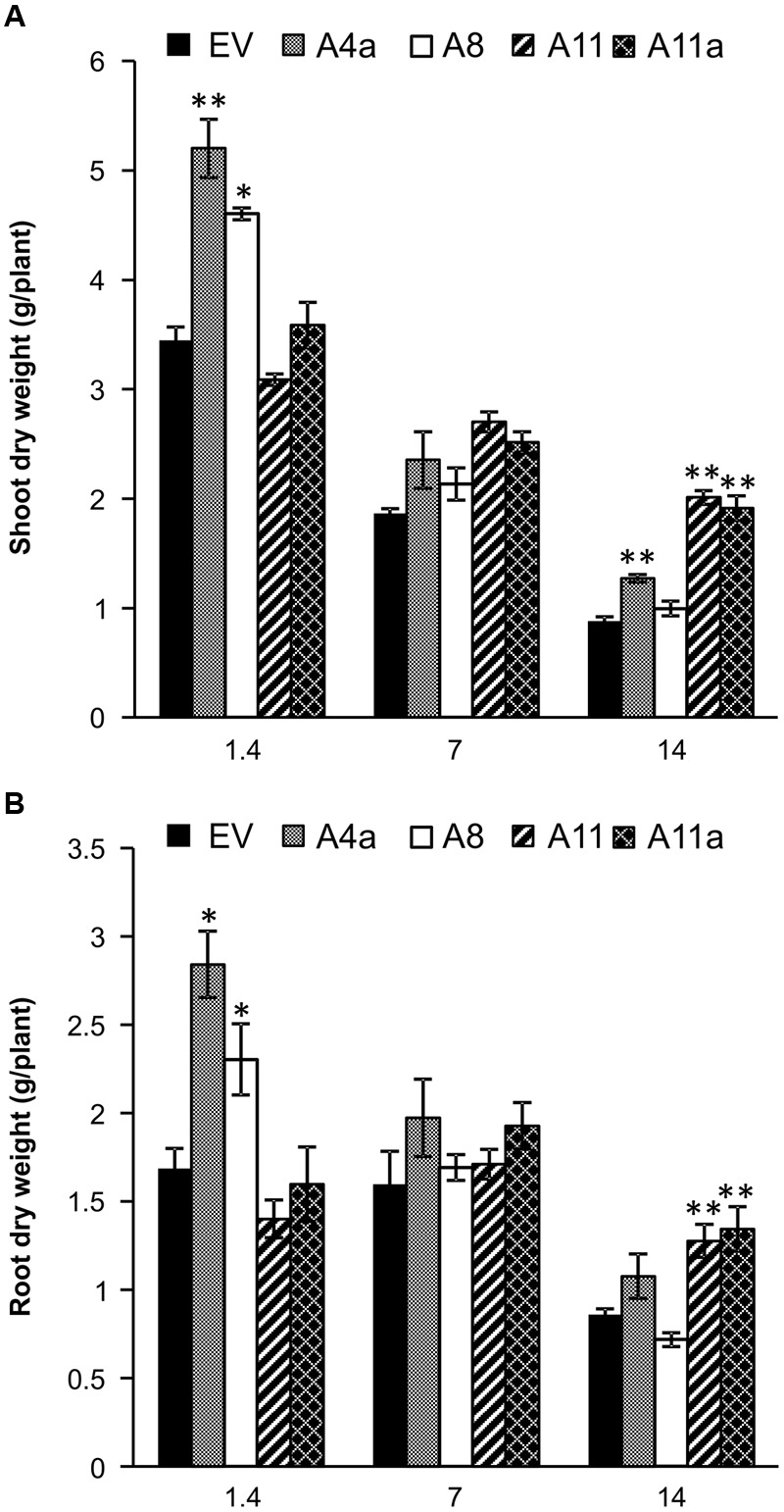
**Dry biomass accumulation in (A)** shoot and **(B)** root of empty vector (EV) and miR156OE genotypes under control, mild and severe salinity stress conditions. Data are average of 6–12 biological replications from each genotype at each stress level. Single asterisk (^∗^) shows significance at *P* < 0.05 and double asterisk (^∗∗^) indicates *P* < 0.01 (ANOVA) between EV and miR156OE genotypes within each stress level (control; EC = 1.4 dSm^-1^, mild; EC = 7 dSm^-1^, severe; EC = 14 dSm^-1^).

Transgenic alfalfa genotypes exhibited variable levels of miR156 expression with A4a and A8 showing low expression, A11a medium and A11 higher expression of miR156 (Supplementary Figure 1A). In our study, alfalfa genotypes overexpressing miR156 accumulated significantly higher shoot dry biomass at least in two miR156OE genotypes (A4a, A8) compared to EV under control (EC 1.4). Three miR156OE genotypes (A4a, A11, and A11a) showed enhanced shoot biomass under severe salt stress (EC 14), while no significant difference was observed under mild salt stress (**Figure 1A**). Similar to leaves, significantly enhanced root dry biomass was observed in A4a and A8 under control conditions compared to EV (**Figure 1B**). Medium and high miR156 over-expressers (A11a, A11) exhibited significantly higher accumulation of dry root biomass compared to EV under severe stress conditions (**Figure 1B**). Furthermore, we observed an increased (statistically or/and numerically) fresh shoot and root biomass accumulation in miR156OE genotypes (except in A8 during severe stress) under control, mild and severe stress conditions (Supplementary Figure 4). We also compared shoot and root biomass gain in EV and miR156OE genotypes during mild and severe stress relative to their corresponding control plants, and results indicated that medium and high miR156 over-expressers (A11, A11a) consistently accumulated biomass that was significantly higher than EV. Low miR156 over-expressers, however, showed insignificant results with EV (Supplementary Figure 5). These results suggest that miR156 improves fresh and dry biomass in alfalfa under control and salinity stress conditions.

### MicroRNA156 Positively Regulates Physiological Responses of Alfalfa under Salinity Stress

**FIGURE 2:**
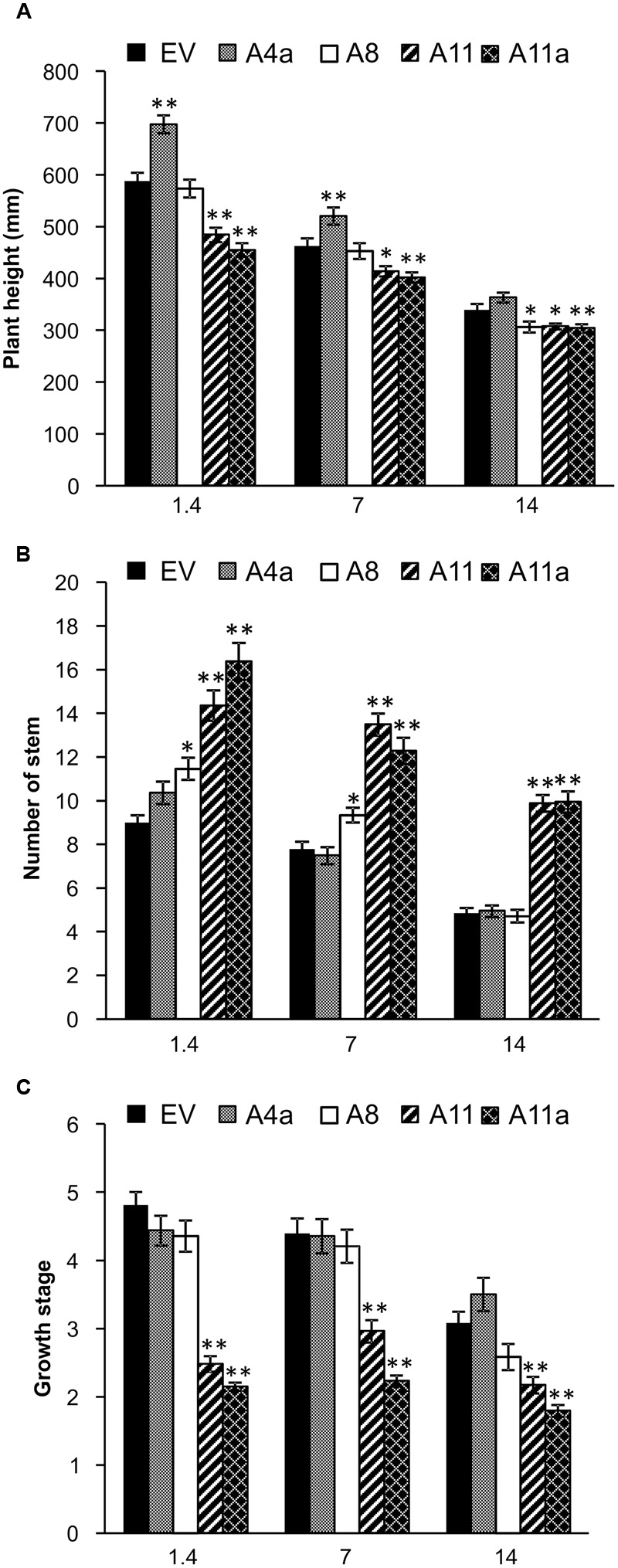
**Physiological traits affected by miR156 overexpression under salinity stress. (A)** Plant height, **(B)** stem number, and **(C)** growth stages. Data are average of four harvests where *n* = 12 for each harvest at each stress level (total 48 biological replications for each genotype at each stress level). Single asterisk (^∗^) shows significance at *P* < 0.05 and double asterisk (^∗∗^) indicates *P* < 0.01 (ANOVA) between EV and miR156OE genotypes within each stress level (control; EC = 1.4 dSm^-1^, mild; EC = 7 dSm^-1^, severe; EC = 14 dSm^-1^).

Plant physiological responses to salinity stress vary and depend on underlying molecular mechanisms (Ahmed et al., 2013). In our study, we measured plant height, number of stems or branches and plant developmental stages in EV and miR156OE genotypes under control, mild and severe salt stress conditions. Results revealed that under control conditions, low miR156 over-expressing plants were either significantly taller (A4a) or showed a similar height compared with EV (A8), while medium and high miR156 over-expressers (A11 and A11a) were significantly shorter. A similar trend was observed in these genotypes under mild stress conditions (**Figure 2A**). Under severe stress, we recorded the same plant height for A4a and EV, whereas A8, A11 and A11a genotypes displayed a significantly shorter height than EV (**Figure 2A**). Interestingly, A11 and A11a genotypes gained significantly more height than EV under mild and severe salt stress conditions when data were presented relative to counterpart plants grown under control conditions (Supplementary Figure 6A), indicating an enhanced ability of medium and high miR156 over-expressers to maintain growth under salinity stress.

Number of stems, which impacts biomass yield, was significantly higher in A8, A11, and A11a than in EV under control and mild stress conditions. Furthermore, medium and high miR156 over-expressers (A11, A11a) showed a significantly higher number of stems compared to EV under severe salt stress, whereas under similar conditions, low miR156 over-expressers (A4a, A8) showed no differences with EV (**Figure 2B**). When severe stress results were compared relative to the corresponding controls, A11a and A11 exhibited significantly higher number of stem compared to EV (Supplementary Figure 6B), providing additional evidence that genotypes with higher levels of miR156 possess better ability to withstand salinity and maintain stem growth.

To further investigate the physiological responses, we recorded developmental stages in EV and miR156OE genotypes. At the time of data collection, medium and high miR156 over-expresser (A11, A11a) were between developmental stages 2 and 3, which was significantly lower than EV and low miR156 expressers (A4a, A8) that were already between stages 4 and 5 under control and mild stress conditions (**Figure 2C**). Similar results were observed under severe stress where A11 and A11a were at or below stage 2 (significantly lower than EV) and low miR156 expressers were at or above stage 3 (**Figure 2C**). Low miR156 expressers (A4a and A8) exhibited developmental stages that were similar to EV under control, mild and severe stress conditions (**Figure 2C**). These results indicate that higher expression of miR156 reduces plant height, increases number of branches and delays developmental phases under control and salt stress conditions. To our surprise, medium and high (A11, A11a) miR156 expressers completed developmental stages significantly faster than EV under severe salt stress conditions when results were presented relative to the corresponding unstressed control plants (Supplementary Figure 6C). This suggested a positive role of miR156 in maintenance of alfalfa physiology and development under salinity stress conditions. Taken together our results show that the ability of alfalfa plants to maintain stem growth and biomass under salinity stress improves with increasing miR156 expression.

### MicroRNA156 Affects Alfalfa Forage Quality and Antioxidant Levels under Salinity Stress Conditions

**FIGURE 3:**
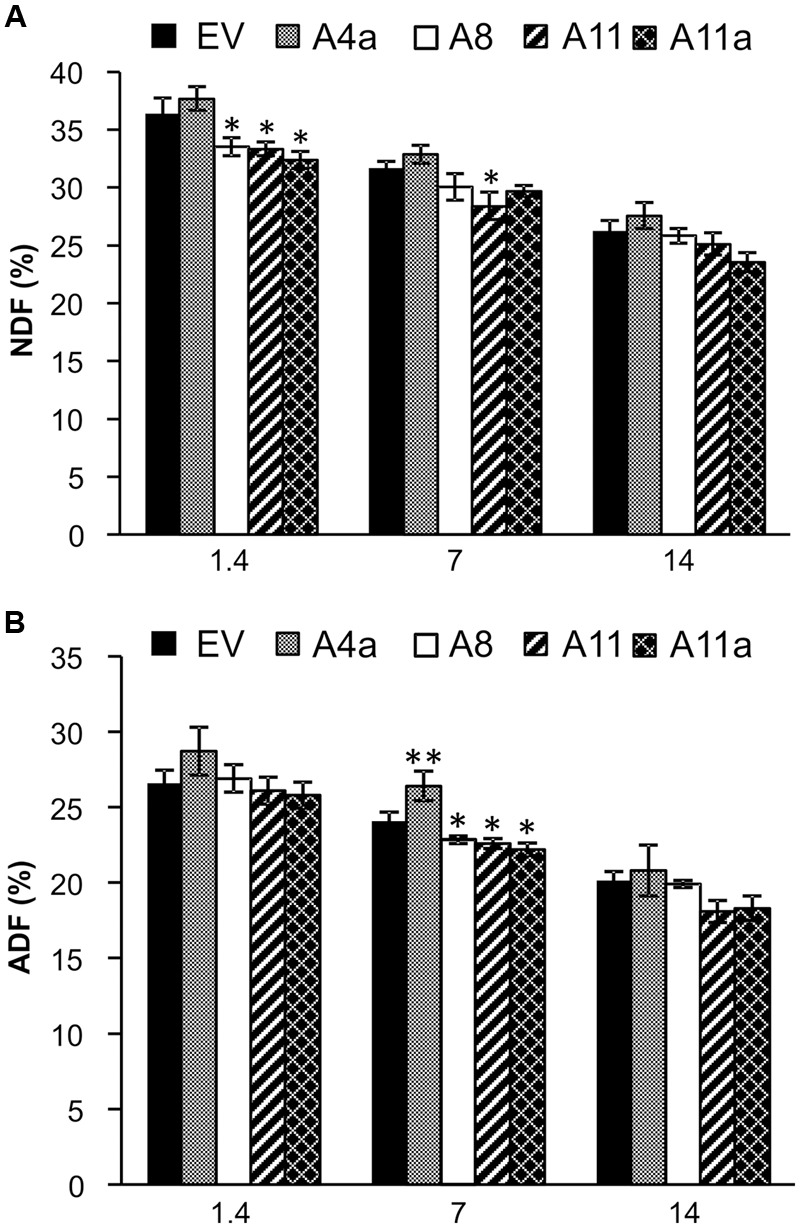
**Effects of miR156 on fiber levels under salinity stress. (A)** Neutral detergent fiber (NDF) and **(B)** acid detergent fiber (ADF) content. Data are average of three harvests where *n* = 3–4 for each harvest at each stress level (total at least nine biological replications for each genotype at each stress level). Single asterisk (^∗^) shows significance at *P* < 0.05 and double asterisk (^∗∗^) indicates *P* < 0.01 (ANOVA) between EV and miR156OE genotypes within each stress level (control; EC = 1.4 dSm^-1^, mild; EC = 7 dSm^-1^, severe; EC = 14 dSm^-1^).

Neutral detergent fiber (NDF) and ADF represent the fiber in plant cell walls and ultimately determine forage quality. Low fiber content indicates higher feed digestibility and nutritional value for animal consumption (Grant et al., 2014). In this study, we observed significantly lower NDF content in three miR156OE genotypes (A8, A11, and A11a) compared to EV under control conditions (**Figure 3A**). The same three genotypes also showed numerically reduced NDF content during mild and severe salt stress compared to EV but differences were statistically insignificant with EV. NDF content was, however, significantly lower in high miR156 over-expressing genotype (A11) compared to EV during mild stress (**Figure 3A**). We did not observe significant differences of ADF content between EV and miR156OE genotypes under control or severe salt conditions (**Figure 3B**). During mild stress, ADF content was significantly reduced in medium and high miR156 over-expressers (A11, A11a) and also in one low miR156 over-expresser (A8) compared to EV controls. In contrast, one of the low miR156 over-expressers (A4a) exhibited significantly higher content of ADF compared to EV (**Figure 3B**).

**FIGURE 4:**
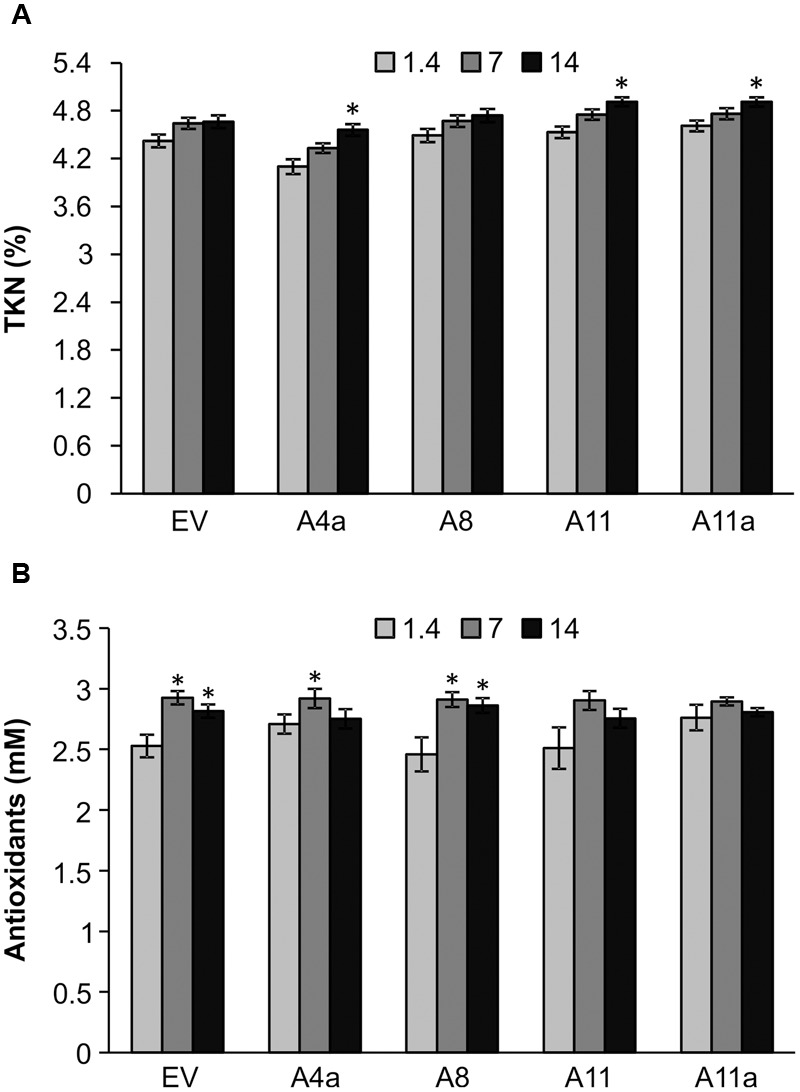
**Nitrogen (A)** and antioxidant **(B)** accumulation in EV and miR156OE plants under salinity stress conditions. Data are average of three harvests where *n* = 4 for each harvest at each stress level for nitrogen (total 12 biological replications for each genotype at each stress level), and one harvest for antioxidants where *n* = 4 for each harvest at each stress level. An asterisk (^∗^) shows significance of mild (EC = 7 dSm^-1^) and severe (EC = 14 dSm^-1^) stress from control (EC = 1.4 dSm^-1^) for each genotype at *P* < 0.05 (ANOVA).

Nitrogen is an essential component of chlorophyll and amino acids and hence plays a crucial role in photosynthesis and protein synthesis. Overall, we did not observe significant differences in TKN between control and mild stress plants of EV and miR156OE genotypes. During severe salinity stress, however, TKN content was significantly increased in A4a, A11, and A11a compared to unstressed counterparts (**Figure 4A**). On comparing EV and miR156OE genotypes, we did not observe differences between EV and three miR156OE genotypes (A8, A11, and A11a) under all conditions. However, a significantly lower level of TKN was observed in A4a compared to EV at control and mild stress (Supplementary Figure 7).

Abiotic stress enhances accumulation of antioxidants in plants to minimize the effect of toxic oxidants produced under stress conditions (Wang et al., 2003). Compared to control plants, mild and severe salt stress slightly induced accumulation of antioxidants in EV and a lower miR156 expresser A8 (**Figure 4B**). On the other hand, another lower miR156 expresser A4a showed increased antioxidant levels only during mild stress compared to corresponding control plants. Furthermore, we did not observe any significant differences in antioxidant accumulation in medium and high miR156 over-expressers (A11, A11a) in response to either mild or severe stress (**Figure 4B**).

Our results reveal that with increasing miR156 expression, nitrogen content also increases whereas fiber content decreases in alfalfa under same salinity conditions, contributing toward improved alfalfa quality as a forage crop.

### MiR156 Modulates Ion Homeostasis under Salinity Stress

To study alfalfa miR156OE genotypes response to ion uptake under saline conditions, we analyzed sodium (Na^+^), calcium (Ca^2+^), zinc (Zn^2+^), and magnesium (Mg^2+^) ions uptake in EV and miR156OE genotypes under control, mild and severe salt stress conditions. Salt stress increased Na^+^ and Zn^2+^ accumulation in EV and four miR156OE genotypes whereas the same conditions reduced Ca^2+^ and Mg^2+^ levels (Supplementary Figure 8). Furthermore, we observed a reduction in Na^+^ uptake at least in medium and high miR156 expressers (A11a, A11) compared to EV under severe salinity stress. Similarly, we also observed reduced levels of Zn^2+^ in A11a during mild stress, and in A8, A11a under severe stress conditions (**Table 1**). On the other hand, a miR156OE genotype (A4a) exhibited higher levels of Ca^2+^ than EV under mild stress. Remaining genotypes showed similar levels of Ca^2+^ and Mg^2+^ compared to EV during mild and severe salinity stress (**Table 1**). These results suggest that alfalfa plants with increased miR156 levels tend to reduce uptake of Na^+^ under severe salt stress conditions.

**Table 1:** Effect of miR156 on ions accumulation in alfalfa.

Genotype	Na^+^	Zn^2+^	Mg^2+^	Ca^2+^
Control (EC = 1.4) ppm				
EV	143.1 ± 16	37.4 ± 4.5	3286 ± 101	21132 ± 493
A4a	128.3 ± 15	30.2 ± 1.7	2810 ± 116	19959 ± 528
A8	135.6 ± 14	35.8 ± 3.8	3184 ± 101	22156 ± 706
A11	171.6 ± 20	36.9 ± 3.7	3244 ± 125	22982 ± 572
A11a	179.8 ± 20	42.2 ± 4.4	3212 ± 103	23192 ± 648
Mild salinity stress (EC = 7 dSm^-1^) – relative to control (EC = 1.4 dSm^-1^)				
EV	987.6 ± 153	150.7 ± 14	74.3 ± 3.3	72.2 ± 3.9
A4a	817.0 ± 109	178.4 ± 29	86.4 ± 4.3	90.8 ± 3.7ˆ*
A8	1049.6 ± 149	166.2 ± 7	76.3 ± 3.5	81.6 ± 3.7
A11	881.6 ± 108	142.3 ± 14	83.9 ± 4.2	82.9 ± 3.2
A11a	848.2 ± 139	119.4 ± 10ˆ*	80.9 ± 3.9	79.9 ± 3.5
Severe salinity stress (EC = 14 dSm^-1^) – relative to control (EC = 1.4 dSm^-1^)				
EV	3230.4 ± 399	208.2 ± 17	89.3 ± 4.7	75.4 ± 5.8
A4a	2749.2 ± 273	205.9 ± 13	96.1 ± 4.7	89.4 ± 6.9
A8	3873.9 ± 472	166.3 ± 7ˆ*	89.9 ± 4.1	82.4 ± 6.7
A11	2219.4 ± 273ˆ*	202.3 ± 20	97.3 ± 4.4	83.2 ± 6.6
A11a	2269.5 ± 332ˆ*	169.8 ± 8ˆ*	92.0 ± 4.3	79.9 ± 5.7

*Ions levels in EV and miR156OE genotypes in response to salinity stress. Values of Na^+^, Ca^*2*+^, Mg^*2*+^, and Zn^*2*+^ at mild (EC = 7 dSm^-*1*^) and severe (EC = 14 dSm^-*1*^) stress are percent of corresponding controls (EC = 1.4 dSm^-*1*^), which is arbitrarily set to 100. Single asterisk (^∗^) shows significance at *P* < 0.05 (ANOVA) between EV and miR156OE genotypes of each ion at each stress level.*

### MiR156 Alters Molecular Responses of Alfalfa under Salinity Stress

To assess the effect of miR156 on molecular responses of alfalfa to salinity, we analyzed expression of genes belonging to three main categories; (1) *SPL* family genes – direct target of miR156, (2) non-SPL transcription factors with known function in salt tolerance and, (3) downstream effector genes with known role in mediating salt responses in plants (see Introduction). We tested the expression of genes in the above-mentioned three categories to determine if miR156 affects these important genes and transcription factors under salt stress for regulation of salinity stress responses in alfalfa.

#### Expression Analysis of miR156-Target SPL Transcription Factors

**FIGURE 5:**
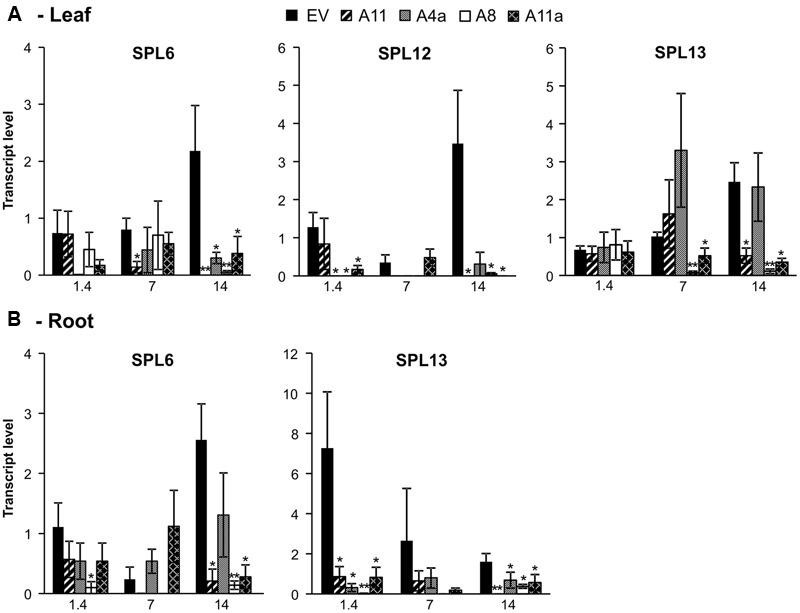
**Relative transcript levels of miR156-target *SPL* genes in leaves (A)** and roots **(B)**. Data are average of 3–4 biological replicates for each genotype at each stress level. Single asterisk (^∗^) shows significance at *P* < 0.05 and double asterisk (^∗∗^) indicates *P* < 0.01 (*t*-test) between EV and miR156OE genotypes within each stress level (control; EC = 1.4 dSm^-1^, mild; EC = 7 dSm^-1^, severe; EC = 14 dSm^-1^).

To assess miR156-regulated molecular responses, we assessed the expression pattern of three miR156-target *SPL* genes (*SPL6, SPL12*, and *SPL13*) (Aung et al., 2015a) in our miR156 over-expressing alfalfa plants. Overall, we observed a consistent pattern of *SPL* gene downregulation in medium and high miR156 expressers under non-stressed and stressed environments compared to low expressers or EV. In leaves, overall expression of *SPL6, SPL12*, and *SPL13* was increased in EV under mild and severe stress conditions whereas expression of these genes was suppressed in all miR156OE genotypes compared to unstressed control (**Figure 5A**). Expression of *SPL6* was significantly reduced in A11 during mild stress and in all miR156OE genotypes under severe stress conditions (**Figure 5A**). Similarly, expression of *SPL12* was significantly reduced in miR156OE genotypes (except A11) and under control and severe stress conditions (except A4a). Mild stress, however, did not cause a significant difference in *SPL12* expression (**Figure 5A**). Furthermore, *SPL13* expression was also significantly reduced in A8 and A11a under mild stress and in A8, A11, and A11a at severe stress compared to EV (**Figure 5A**).

In roots, we observed a significant downregulation of *SPL6* under severe salt stress with three miR156OE genotypes (A8, A11, and A11a) showing reduced expression. More pronounced results were observed in *SPL13* expression, which was significantly downregulated in all miR156OE genotypes compared to EV under control and severe stress conditions (**Figure 5B**). However, we did not detect significant changes of *SPL12* expression in root (data not shown). These results suggest that miR156 targets SPL family more efficiently under salt stress conditions.

#### Expression Analysis of Abiotic Stress Responsive Non-SPL Transcription Factors

**FIGURE 6:**
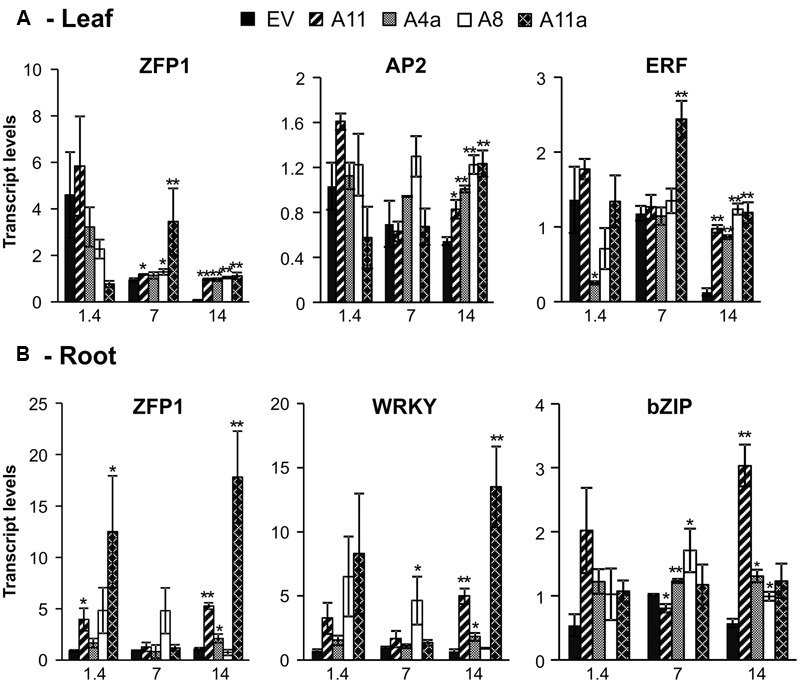
**Relative transcript levels of abiotic stress responsive transcription factors in leaves (A)** and roots **(B)**. Data are average of 3–4 biological replicates for each genotype at each stress level. Single asterisk (^∗^) shows significance at *P* < 0.05 and double asterisk (^∗∗^) indicates *P* < 0.01 (*t*-test) between EV and miR156OE genotypes within each stress level (control; EC = 1.4 dSm^-1^, mild; EC = 7 dSm^-1^, severe; EC = 14 dSm^-1^).

In leaves, transcription factor *ZFP* showed no difference in expression between EV and miR156OE genotypes under control conditions (**Figure 6A**). During mild stress, expression of *ZFP1* was significantly higher in three miR156OE genotypes (A8, A11, and A11a) and, in addition, a strong and significant induction was observed in all miR156OE genotypes compared to EV during severe stress (**Figure 6A**). The *ethylene response factor (ERF)* also showed no expression differences between EV and miR156OE genotypes (except A4a) under control conditions. Upon exposing plants to mild salinity stress, *ERF* expression was similar between miR156OE genotypes (except A11a) and EV. More prominent expression differences were observed under severe stress conditions when miR156OE genotypes showed significant upregulation of *ERF* compared to EV (**Figure 6A**). A similar expression pattern was observed also for *AP2* domain transcription factor (*AP2*), which was significantly induced in miR156OE genotypes under severe salt stress (**Figure 6A**).

In roots, medium and high miR156 expressers (A11, A11a) showed significant upregulation of *ZFP1* compared to EV under control conditions (**Figure 6B**). Under mild stress, all miR156OE genotypes showed expression of *ZFP1* that was similar to EV. More pronounced results were observed during severe stress when three miR156OE genotypes (A4a, A11, and A11a) showed significantly higher expression, and A8 showed similar expression of *ZFP1* compared to EV (**Figure 6B**). *WRKY* and *bZIP* transcription factors exhibited similar expression to miR156OE genotypes and EV under control conditions (**Figure 6B**). Expression of *WRKY* was also similar between EV and miR156OE (except A8) at mild stress, whereas under severe stress, *WRKY* was significantly upregulated in three miR156OE (A4a, A11, and A11a) genotypes. Under mild stress, expression of root *bZIP* was significantly induced in two low miR156 expressers (A8, A4a), significantly lower in a higher expresser (A11), but no different in a medium miR156 expresser (A11a) compared to EV. All miR156OE genotypes showed upregulation of *bZIP* at severe stress with A4a, A8, and A11 showing significant differences compared with EV (**Figure 6B**). Expression differences of *AP2* and *ERF* in roots, and *WRKY* and *bZIP* in leaves were not significant between EV and miR156OE genotypes under control and stress conditions (data not shown). Expression patterns of transcription factors that differ between leaves and roots suggested that miR156 affects transcriptional activities in a tissue-specific manner.

#### Expression Analysis of Downstream Candidate Salt Responsive Genes

**FIGURE 7:**
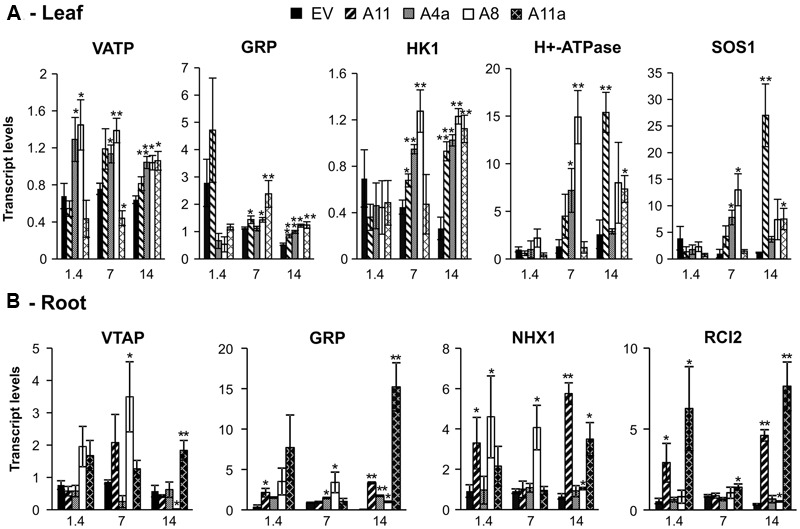
**Relative transcript levels of salt stress responsive genes in leaves (A)** and roots **(B)**. Data are average of 3–4 biological replicates for each genotype at each stress level. Single asterisk (^∗^) shows significance at *P* < 0.05 and double asterisk (^∗∗^) indicates *P* < 0.01 (*t*-test) between EV and miR156OE genotypes within each stress level (control; EC = 1.4 dSm^-1^, mild; EC = 7 dSm^-1^, severe; EC = 14 dSm^-1^).

We further analyzed expression of candidate genes involved in salt stress tolerance such as vacuolar Na^+^/H^+^ antiporter (*NHX*), plasma membrane H^+^-ATPase, cation/H^+^ exchanger 3; a putative homolog of *Arabidopsis* salt overly sensitive 1 (*SOS1*), low temperature and salt responsive family protein 2 (*RCI*2), *Vacuolar H*^+^ *pumping ATPase* (*VATP*), *Glycine rich protein* (*GRP*) and a *cytokinin receptor* homolog (*HK1*). *VATP, GRP, NHX1, RCI2, H*^+^*-ATPase and SOS1* are salt-induced genes, and their over-expression enhanced salinity stress tolerance in plants (Coba de la Pena et al., 2008; Bao et al., 2009; Yang et al., 2009; Leidi et al., 2010; Long et al., 2013). In leaves, transcript levels of *VATP* were significantly increased only in low miR156 expressers, whereas expression levels of *GRP* and *HK1* were insignificant in miR156OE genotypes compared to EV under control conditions (**Figure 7A**). Mild stress, however, induced expression of *VATP* in low miR156 expressers (A4a and A8) while reducing *VATP* transcription in a medium miR156 expresser (A11a) compared to EV. Under mild stress conditions, A11 showed expression of *VATP* that was similar with EV. Furthermore, severe salt stress induced expression of *VATP* in leaves of all miR156OE genotypes, with A8, A4a, and A11a being significantly higher than EV (**Figure 7A**). *GRP* expression was significantly increased in miR156OE genotypes (except A4a) under mild stress, while all miR156OE genotypes exhibited significant upregulation of *GRP* under severe stress conditions (**Figure 7A**). In addition, all miR156OE genotypes showed significant upregulation of *HK1* under mild (except A11a) and severe salt stress compared to EV (**Figure 7A**). Similarly, *H*^+^*-ATPase* and *SOS1* showed similar expression trends in leaves where low miR156 expressers (A8, A4a) exhibited higher expression during mild stress, and A11 and A11a showed higher expression under severe salt stress compared to EV (**Figure 7A**).

In roots, expression of *GRP, NHX1*, and *RCI2* was significantly or insignificantly higher in miR156OE genotypes compared to EV control. We also did not observe a significant difference in expression of *VATP* between EV and miR156OE genotypes under control and mild stress conditions (except in A8 under mild stress). However, during severe stress *VATP* expression was significantly induced in A11a but reduced in A8, while remaining similar in A4a and A11 compared to EV (**Figure 7B**). Compared to EV, all miR156OE genotypes showed higher expression of *GRP* under severe stress conditions, while A11 showed higher expression only under non-saline conditions and A4a and A8 under mild stress (**Figure 7B**). The miR156OE genotypes showed higher expression of *NHX1* even under non-saline conditions, with A8 and A11 having significantly higher expression than in EV (**Figure 7B**). During mild stress, only A8 showed significantly higher expression of *NHX1* whereas under severe stress, A8, A11, and A11a showed significantly higher expression compared to EV (**Figure 7B**). Similarly, medium and high expressers (A11, A11a), showed higher expression of *RCI2* compared to EV under control conditions. Under mild stress, only A11a exhibited upregulation of *RCI2* compared to EV whereas under severe stress conditions, three miR156OE genotypes (A8, A11, and A11a) exhibited upregulation of this genes compared to EV (**Figure 7B**). We did not observe significant differences in expression of *NHX1* and *RCI2* in leaves, and *HK1, H*^+^*-ATPase*, and *SOS1* in roots of miR156OE genotypes and EV (data not shown).

These results suggest that miR156 affects expression of candidate salt responsive genes in a tissue-specific manner under stress conditions. Importantly, we observed more pronounced and consistent results in medium and high miR156 expressers (A11, A11a) under severe salinity stress (EC 14).

## Discussion

Salinity is a serious threat to growth and productivity of alfalfa and other crops around the world. In addition, climate change may intensify abiotic stress conditions including salinity in alfalfa growing regions, particularly in the North American prairies. Salt tolerant genotypes have different strategies to cope with stress conditions such as slowing down growth, synthesizing defense compounds and antioxidants, altering gene expression and maintaining ion homeostasis. In this study, we dissected the role of miR156 in salinity tolerance of alfalfa. Our results suggest that transgenic alfalfa plants with medium to high miR156 expression (A11a, A11) exhibit consistent and robust responses under severe salinity stress (EC 14 dSm^-1^) compared to the genotypes with low miR156 expression levels (A4a, A8), and under medium stress (EC 7 dSm^-1^) or control (EC 1.4 dSm^-1^) conditions.

### Improved Biomass and Forage Quality under Salinity Stress

Alfalfa biomass is an important trait for forage and biofuel industries. In our study, miR156 overexpressing plants exhibited an increase in shoot and root biomass under control conditions as previously reported (Aung et al., 2015a). Normally, salinity reduces plant biomass accumulation (Li et al., 2010) but, interestingly, we recorded a less reduction in biomass accumulation in miR156OE genotypes relative to EV under severe stress condition. In absolute term, an increased branching (shoot number) and slower developmental stage completion by miR156OE genotypes compared to EV may have contributed to the accumulation of higher biomass. Surprisingly, miR156OE genotypes were quick in completing developmental stages, and also exhibited increased plant growth (height, stem, and developmental stages) in relation to their corresponding controls, when compared with EV under stress conditions, indicating a stress avoidance mechanism in transgenic alfalfa plants under salinity stress. Previously, an increased shoot growth was observed in tomato and rice under salt stress (Zhu et al., 1998; Mayak et al., 2004).

Total Kjehldahl nitrogen (TKN), NDF, and ADF contribute to forage quality and digestibility. Protein increases forage quality, and reduction in nitrogen content leads to reduced crude protein content, which ultimately reduces forage quality (Owensby et al., 1996). NDF consists of cellulose, hemicellulose and lignin whereas ADF contains mainly cellulose and lignin but lacks hemicellulose (Van Soest, 1994). Furthermore, NDF and ADF are components of fiber that can reduce digestibility, palatability, and intake of forages leading to degradation of forage quality (Van Soest, 1994). Our study shows a significant increase of TKN in response to severe salt stress in three miR156OE genotypes but not in EV plants. Moreover, we recorded a reduction of NDF and ADF in miR156OE genotypes compared to EV under control and mild salt stress conditions, respectively. An increased TKN and decreased NDF and ADF content in miR156OE genotypes indicates that miR156 also contributes to alfalfa forage quality improvement under normal and saline conditions. A study has shown an improved forage quality of bermudagrass and wheatgrass under salinity stress (Robinson et al., 2004). Similarly, in another study miR156 overexpression led to enhanced forage quality in switchgrass (Fu et al., 2012).

### Molecular Responses and Ion Homeostasis in Transgenic Plants under Salinity Stress

Higher levels of Na^+^ ions can cause ion toxicity resulting in reduced plant growth and performance. Several membrane transporters particularly Na^+^ and K^+^ transporters play a crucial role in salinity tolerance in plants (Schroeder et al., 2013). Most studies have been conducted in *Arabidopsis* where cytokinin mediates expression of *AtHKT1* in a negative manner via type B response regulators ARR1 and ARR12, thereby indicating that cytokinin levels are reduced in response to salinity stress, resulting in induction of *AtHKT1* expression (Mason et al., 2010), which supports our *HK1* expression results.

Previously, studies have identified a group of *HKT* transporters that regulate Na^+^/K^+^ co-transport (Rubio et al., 1995). Subsequent studies have suggested that AtHKT1-mediated Na^+^ removal from the xylem stimulates K^+^ loading into xylem, resulting in a high K^+^/Na^+^ ratio in leaves that also alleviates Na^+^ stress (Ren et al., 2005; Sunarpi et al., 2005). There is an additional study showing that HKTs play a crucial role in determining a high K^+^/Na^+^ ratio in plants. For example, a *TaHKT1* gene contributed to Na^+^ removal from xylem in the leaf sheath of durum wheat to protect leaf blades from Na^+^ over accumulation and toxicity (Huang et al., 2006). Mutating *Arabidopsis HKT* gene in *(AtHKT1)* caused Na^+^ hypersensitivity, as well as enhanced accumulation of Na^+^ upon salinity stress in *Arabidopsis* leaves (Mäser et al., 2002; Berthomieu et al., 2003; Horie et al., 2006). Subsequent studies further demonstrated the role of *AtHKT1*, and a rice ortholog *OsHKT1*, in removing Na^+^ and protecting leaves from Na^+^ toxicity (Ren et al., 2005; Sunarpi et al., 2005; Davenport et al., 2007). Another study conducted on root stellar cells in *Athkt1* mutant plants provided additional evidence that *AtHKT1* mediates passive Na^+^ channel-like transport (Xue et al., 2011).

In our study, salt stress induced Na^+^ accumulation in EV and all miR156OE genotypes, but this accumulation was reduced in moderate and high miR156 expressers (A11, A11a) compared to EV under severe stress conditions. Reduced accumulation of Na^+^ in A11 and A11a could be due to induced expression of *HK1* in these genotypes under severe stress since *HKT* transporters play key role in Na^+^ efflux from the xylem (Sunarpi et al., 2005). Furthermore, tonoplast and plasma membrane localized *NHXs* antiporters are essential for maintaining low Na^+^ in the cytoplasm and Na^+^ detoxification via sequestering within the vacuole (Deinlein et al., 2014). Over-expression of *NHX* led to salinity tolerance in *Arabidopsis* (Apse et al., 1999). A recent transcriptome study revealed that alfalfa transporter genes *HKT1* and anion exchanger maintained high transcript levels in salt tolerant alfalfa genotypes (Gruber et al., 2016). Na^+^ influx can raise sodium concentration inside the cytosol activating K^+^ efflux and disturbing K^+^/Na^+^ ratio (Sun et al., 2009; Britto et al., 2010). The high-affinity K^+^ transporter (*HKT*) family transports Na^+^ and K^+^ (Benito et al., 2014). Our results showed reduced Na^+^ concentration in medium and high miR156 expressors (A11, A11a) under severe stress, which may have increased K^+^ influx. This is also supported by induced expression of *HK1* in leaves of miR156OE genotypes. Overexpression of *Arabidopsis H*^+^*-ATPase* and *SOS1* enhances salt tolerance in plants (Bao et al., 2009; Yang et al., 2009). In our study, an enhanced expression of *NHX1* in root and *HK1, H*^+^*-ATPase and SOS1* in leaves of medium and higher miR156 expressers under severe saline conditions may have contributed to the removal of Na^+^ and a reduction of its toxicity in these genotypes.

Salinity stress induces antioxidant accumulation in plants to counter the damage caused by oxidants under stress conditions (Bose et al., 2014). In our study, we did not observe a slight induction of antioxidants in medium and higher expressers of miR156 (A11, A11a) under mild or severe stress. This may indicate that these genotypes may have low internal stress level compared to EV and low miR156 expressers (A4a, A8) that accumulated significantly higher levels of antioxidants in response to mild and severe stress.

**FIGURE 8:**
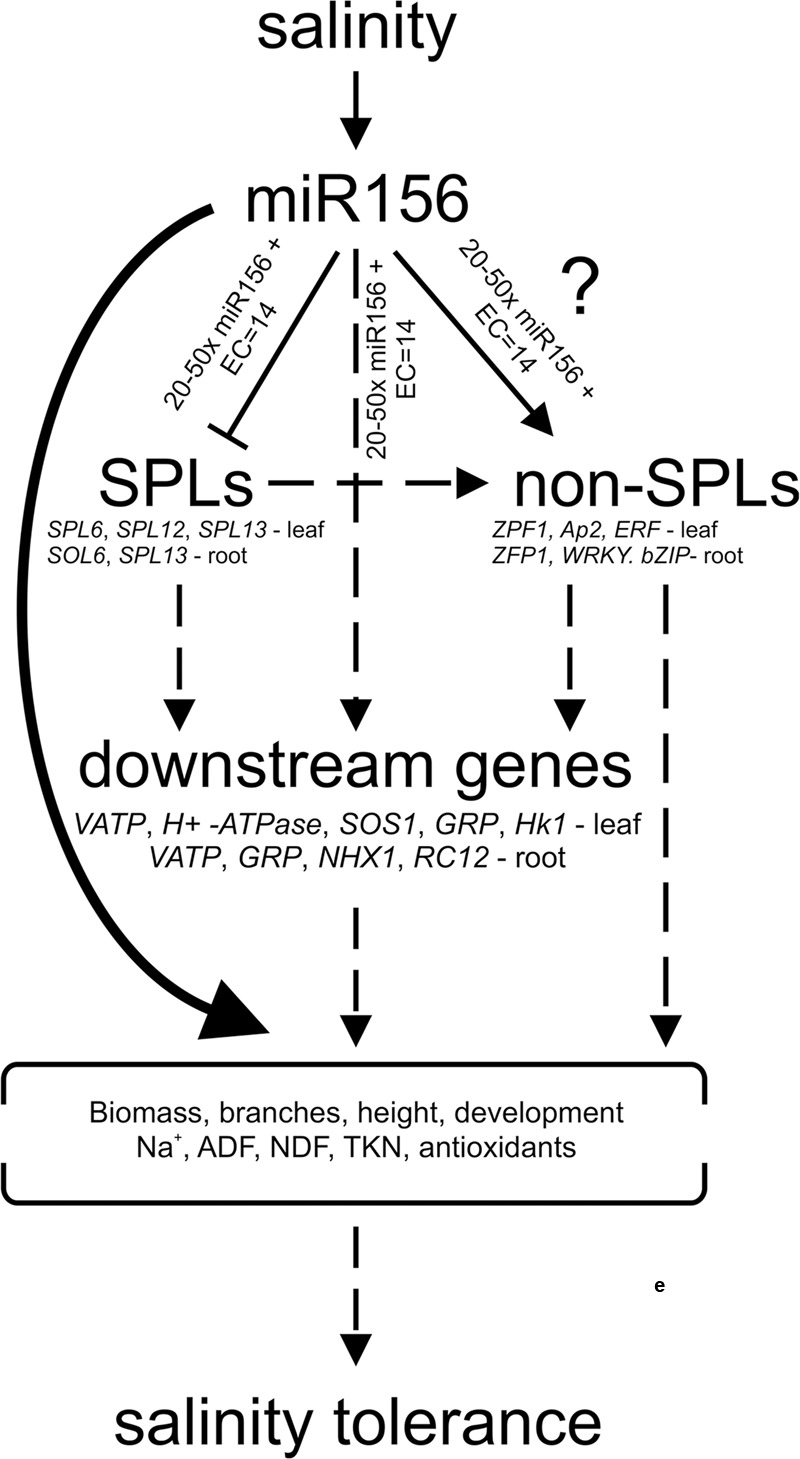
**A model showing pathways for salinity tolerance in alfalfa.** Solid lines or arrows indicate a pathway supported by experimental evidence or reports from literature. Dotted lines or arrows show a suggested pathway.

Given that SPL transcription factors (SPL6, SPL12, and SPL13) are targeted by miR156 (Aung et al., 2015a), it is important to analyze their expression patterns to better understand the mechanism of salt tolerance regulated by miR156. Previously, there was one study that revealed that rSPL9 (expressing miR156-insensitive *SPL9*) and rSPL10 (expressing miR156-insensitive *SPL10*) plants exhibited hypersensitivity to drought and salt stress (Cui et al., 2014). Their results indicate that SPLs could potentially be negative regulators of stress response. Similarly, genes encoding non-SPL transcription factors such as *ZFP1, AP2, ERF, WRKY*, and *bZIP* and other downstream effector genes play vital role in salt tolerance in plants (Singh et al., 2002; Ciftci-Yilmaz and Mittler, 2008; Jiang and Deyholos, 2009; Pandey and Somssich, 2009; Rushton et al., 2010; Mizoi et al., 2012; Shi et al., 2014; Hu et al., 2016), and testing expression of these genes could also be helpful in expanding list of direct or indirect targets of miR156 particularly under salt stress. This information could be helpful in drawing a pathway that miR156 uses to regulate salinity stress responses in alfalfa (**Figure 8**).

Several studies have established that miR156 modulates abiotic stress responses in various plant species (Wang M. et al., 2013; Cui et al., 2014; Kohli et al., 2014; Stief et al., 2014; Xie et al., 2014; Wang and Wang, 2015). MiR156 controls plant responses through targeting *SPL* family transcription factors (Aung et al., 2015a), which in turn may alter expression of downstream genes. We observed downregulation of *SPL* genes in leaves and roots under stress conditions indicating that miR156 expedites the cleavage of *SPLs* to modulate stress responses in alfalfa. *SPL* genes respond to abiotic stress and stimulate the synthesis of secondary metabolites (Wang M. et al., 2013; Yu et al., 2015) showing that they may directly or indirectly be involved in stress tolerance.

Under salt stress conditions, several genes and transcription factors including *WRKY, ZFP, bZIP*, and *ERF/AP2* are upregulated to help plants adapt to saline conditions (Golldack et al., 2011; Deinlein et al., 2014). In our study, however, not all the tested genes and transcription factors were upregulated in response to salt stress. An enhanced expression of *WRKY* and *ZFP1* in leaf and root, *ERF* and *AP2* in leaf, and *bZIP* in root of miR156OE genotypes under salt stress indicates that transcription activities were increased in miR156OE genotypes. We hypothesize that as transcription activators, *SPLs* may have an inverse relationship with other stress responsive transcription factors (Gou et al., 2011), and a reduced expression of *SPLs* may have led to enhanced transcriptional activities of *WRKY, ZFP, bZIP*, and *ERF/AP2* transcription factors in leaves and roots of stressed alfalfa plants.

Overexpression of downstream salt responsive genes such as *NHX1, VATP, GRP, HK1 RCI2, H*^+^*-ATPase*, and *SOS1* improve salt stress tolerance in plants (Coba de la Pena et al., 2008; Silva and Geros, 2009; Leidi et al., 2010; Long et al., 2013, 2015). In our study, upregulation of these genes in leaf and root of miR156OE genotypes under salt stress conditions may have contributed to salt stress tolerance of transgenic alfalfa. This also provides further support to our hypothesis that high transcription activities in miR156OE plants led to altered expression of downstream genes resulting in an adaptive response of alfalfa plants to salt stress.

Overall, three common tendencies can be found in our data; (1) tissue-specific –in case of *SPL12, ERF, AP2, GRP, HK1*, (show expression differences only in leaf) and *WRKY, bZIP, NHX1, RCI2* (show differences only in root), (2) severe stress-specific – show consistent downregulation of SPLs, and upregulation of non-SPLs transcription factor as well as downstream effector genes under severe stress conditions across leaf and root, and (3) miR156 level-specific – show consistently significant results in medium and high miR156 expresssors (A11, A11a). This supports our hypothesis that a relationship exists between miR156 levels, SPLs, non-SPL transcription factors and downstream genes.

Taken together, we report that miR156 positively regulates salinity response in alfalfa partially by altering expression of important transcription factors and downstream genes. Thus, a trend seems to exist where increased expression of miR156 exhibits a salt tolerant phenotype during severe stress. We noted that gene expression in plants is differently regulated and depends on the intensity of stress and the tolerance or sensitivity of the genotypes. Our results are in line with other published studies. For example, a study by (Rodrigues et al., 2009) showed an insignificant expression of several genes at mild water stress in sugarcane cultivars, but their expression was significantly increased under severe stress. In support of genotypic differences, (El Maarouf et al., 1999) observed that a susceptible cowpea genotype showed enhanced expression of *Phospholipase D* under drought stress conditions whereas a tolerant genotype showed low expression of this gene throughout the experiment. These studies support our results of variable expression in different genotypes at different stress levels.

Several studies in other plant species have revealed that miR156 levels do not only change in response to salt stress but also under various other stresses such as heat, drought, cold, UV-B radiation, and hypoxia (Zhou et al., 2008; Jia et al., 2009; Moldovan et al., 2010; Cui et al., 2014; Stief et al., 2014). However, we cannot rule out the possibility that salt stress response is in part regulated by additional salt-responsive genes and transcription factors not identified in this study, which may directly or indirectly be regulated by miR156. Whereas this study reveals a role for miR156 in salt stress response, further studies involving next generation sequencing, and functional characterization of target *SPL* genes and downstream SPL-regulated genes would be needed to fully understand how miR156 affects salt tolerance. Our recent study has shown *SPL13* as a negative regulator of drought responses in alfalfa (Arshad et al., 2017), and a similar work under salt stress would provide a better understanding of how the miR156-SPL gene regulatory network affects salt tolerance. The detailed analysis of the regulation of miR156, and other salt-responsive genes and transcription factors remains an important area of research, as it will be interesting to see whether miR156 along with identified genes and transcription factors also play a role in improving tolerance to other stresses in various plant species.

## Conclusion

We propose that high miR156 levels (20–50 times) result in a robust and consistent response to salinity stress in alfalfa. Similar miR156 levels improve growth, biomass and forage quality of alfalfa during salinity stress. Elevated levels of miR156 reduce the uptake of toxic ions and increase beneficial ions, a condition which may contribute to salinity tolerance. In addition, we hypothesize that miR156 directly or indirectly targets transcription factors including SPLs, which in turn may regulate downstream genes in a stress-specific manner leading to salinity stress tolerance in alfalfa. These transcription factors and downstream affected genes could be suitable targets for alfalfa breeding programs.

## Author Contributions

MA conducted experiments, collected, analyzed and interpreted data, as well as drafted manuscript. MG conducted experiments, collected data, and edited manuscript. KW conducted experiments and collected data. AH secured funding, oversaw the project and edited manuscript. All authors critically reviewed and approved the final manuscript.

## Conflict of Interest Statement

The authors declare that the research was conducted in the absence of any commercial or financial relationships that could be construed as a potential conflict of interest.
