# Supplementary material for: An Insight into microRNA156 Role in Salinity Stress Responses of Alfalfa
Source: Front Plant Sci. 2017 Mar 14;8:356. doi: 10.3389/fpls.2017.00356 (PMC5348497; doi:10.3389/fpls.2017.00356)
Supplement: Supplementary file 1 [file Table_1.DOCX]

**Supplementary table 1.** List of primers used for qRT-PCR analysis. Primers were designed from *M. sativa* sequences.

| Gene name | Primer sequence | *M. truncatula* homolog |
| --- | --- | --- |
| Acetyl CoA Carboxylase (*ACC1*) | \| GATCAGTGAACTTCGCAAAGTAC \| \| --- \| \| CAACGACGTGAACACTACAAC \| | *Medtr3g073860.1* |
| *Actin* | \| AGCAAAAGATGGCAGATGCT \| \| --- \| \| CCATACCAACCATGACACCA \| | *Medtr3g095530.3* |
| *SQUAMOSA PROMOTER BINDING*  *PROTEIN-LIKE 6 (SPL6)* | TGACAAGCTCCGCGAATAAG  AACAGTGGCACCATGTTCAG | *Medtr5g046670.1* |
| *SQUAMOSA PROMOTER BINDING*  *PROTEIN-LIKE 12 (SPL12)* | CCCCCAAACCAAAGATTTTA   \| TCTTGGTTCCTTTGCCTTTG \| \| --- \| | *Medtr3g085180.1* |
| *SQUAMOSA PROMOTER BINDING*  *PROTEIN-LIKE 13 (SPL13)* | CCAGGTGCTACCCTTTTCAA  CATGAACTTGCTGGTGTTGG | *Medtr3g099080.1* |
| *APETALA2/ETHYLENE-RESPONSIVE FACTOR (AP2/ERF) - ERF* | \| TTTGGAAGCTGGTGTTTGTG \| \| --- \| \| CATCACCAGCAACTGATTGG \| | *Medtr1g040430.1* |
| *APETALA2/ETHYLENE-RESPONSIVE FACTOR (AP2/ERF) - AP2* | \| TCCTTCAACAAACACCACCA \| \| --- \| \| TCCTTCAACAAACACCACCA \| | *Medtr5g009410.1* |
| *WRKY* | \| GGTGGAGCAACCTCTAACCA  TCAGCGCCTTCTTTTGTCTT \| \| --- \| | *Medtr7g079040.1* |
| *ZINC FINGER PROTEIN 1 (ZPF1)* | \| TCCTTCATTTGCAAGCCTTT \| \| --- \| \| TCCTTCATTTGCAAGCCTTT \| | *Medtr4g006575.1* |
| *VACUOLAR H^+^ PUMPING ATPASE (VATP)* | \| TGCAACTGGTGTCCAAACAT \| \| --- \| \| GGCCAGTCTAGAGCCGAGTA \| | *Medtr4g072050.1* |
| *GLYCINE-RICH PROTEIN (GRP)* | \| GCTTCCTCATCTTTGGCAAC \| \| --- \| \| AATTCCACCAACACCTGGAA \| | *Medtr2g012520.1* |
| *CYTOKININ RECEPTOR HOMOLOGUE (HK1)* | \| ATTCAGAAATCTCAGCTCAGAG  GAA \| \| --- \| \| AGACTTTTCATACCCTCACAAC  CAT \| | *Medtr3g085130.1* |
| *BASIC REGION/LEUCINE ZIPPER MOTIF (bZIP)* | \| TGCTTCCCCAATGTTGAAGA \| \| --- \| \| TCTGTGCTTGACGTCGTAGT \| | *Medtr5g060950.1* |
| *VACUOLAR NA+/H+ ANTIPORTER 1 (NHX1)* | \| TCCCACCCATCATTTTCAAT \| \| --- \| \| CTCCAATCTTGAGGGAACCA \| | *Medtr4g118770.1* |
| *RARE COLD INDUCIBLE 2 (RCI2)* | \| TGACCAAACCAAACTGTGGA \| \| --- \| \| GTGGAGTGGACCATCGAACT \| | *Medtr7g111380.1* |
| *PLASMA MEMBRANE H+-ATPASE (H+-ATPase)* | GCTCTTATGGCTGGTCTTGC  CCGCTGGAACAATATCACCT | *Medtr4g127710.1* |
| *CATION/H+ EXCHANGER 3; a putative homolog of Arabidopsis Salt Overly Sensitive 1 (SOS1)* | AGCGCATTCTCAATGGAAGT  CCAGCCATACGGGAAAGTAA | *Medtr2g038400.1* |
