# Supplementary material for: An Insight into microRNA156 Role in Salinity Stress Responses of Alfalfa
Source: Front Plant Sci. 2017 Mar 14;8:356. doi: 10.3389/fpls.2017.00356 (PMC5348497; doi:10.3389/fpls.2017.00356)
Supplement: Supplementary file 2 [file Presentation_1.PPTX]

## Slide 1
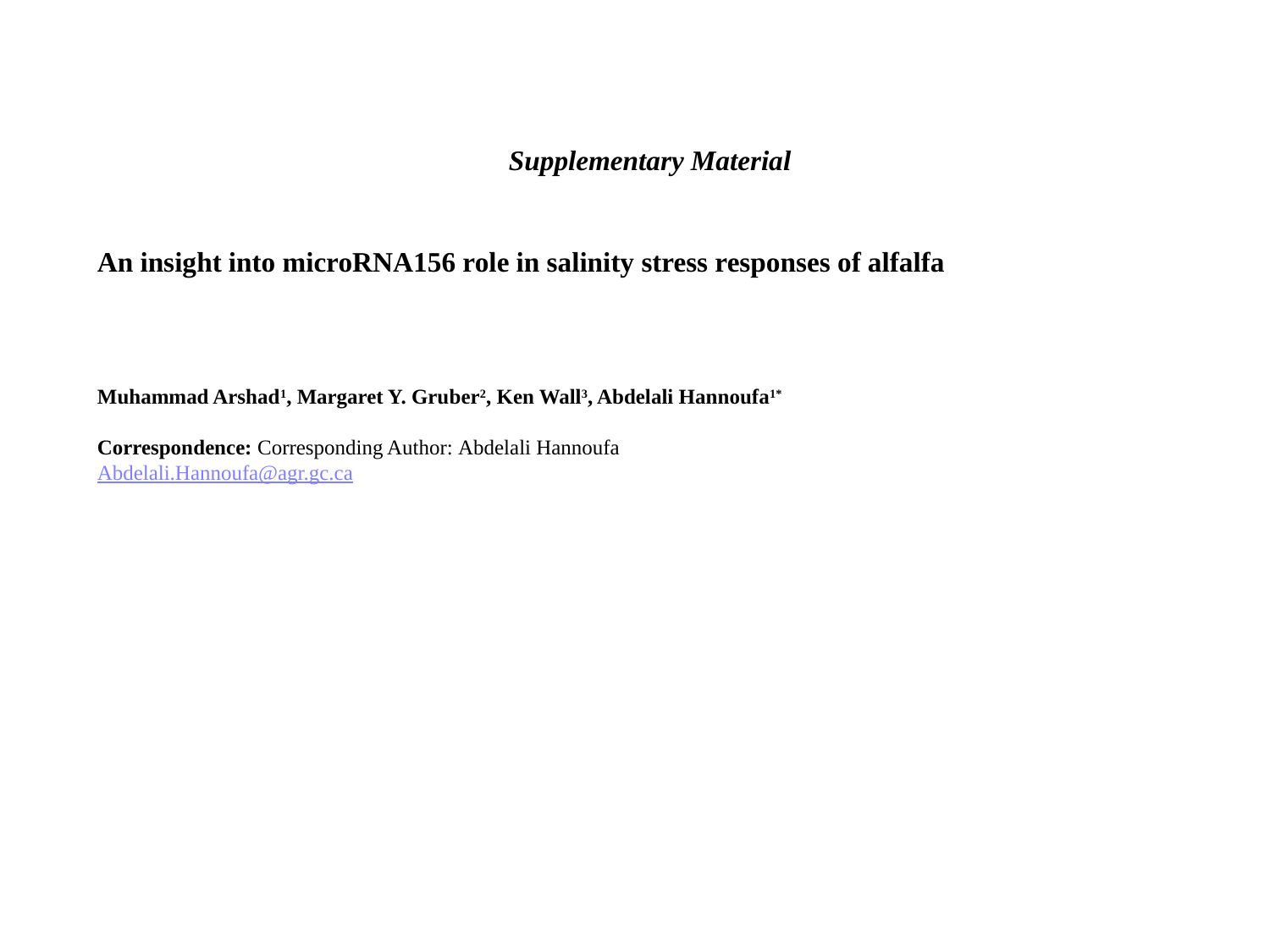

Supplementary Material
An insight into microRNA156 role in salinity stress responses of alfalfa
Muhammad Arshad1, Margaret Y. Gruber2, Ken Wall3, Abdelali Hannoufa1*
Correspondence: Corresponding Author: Abdelali Hannoufa
Abdelali.Hannoufa@agr.gc.ca

## Slide 2
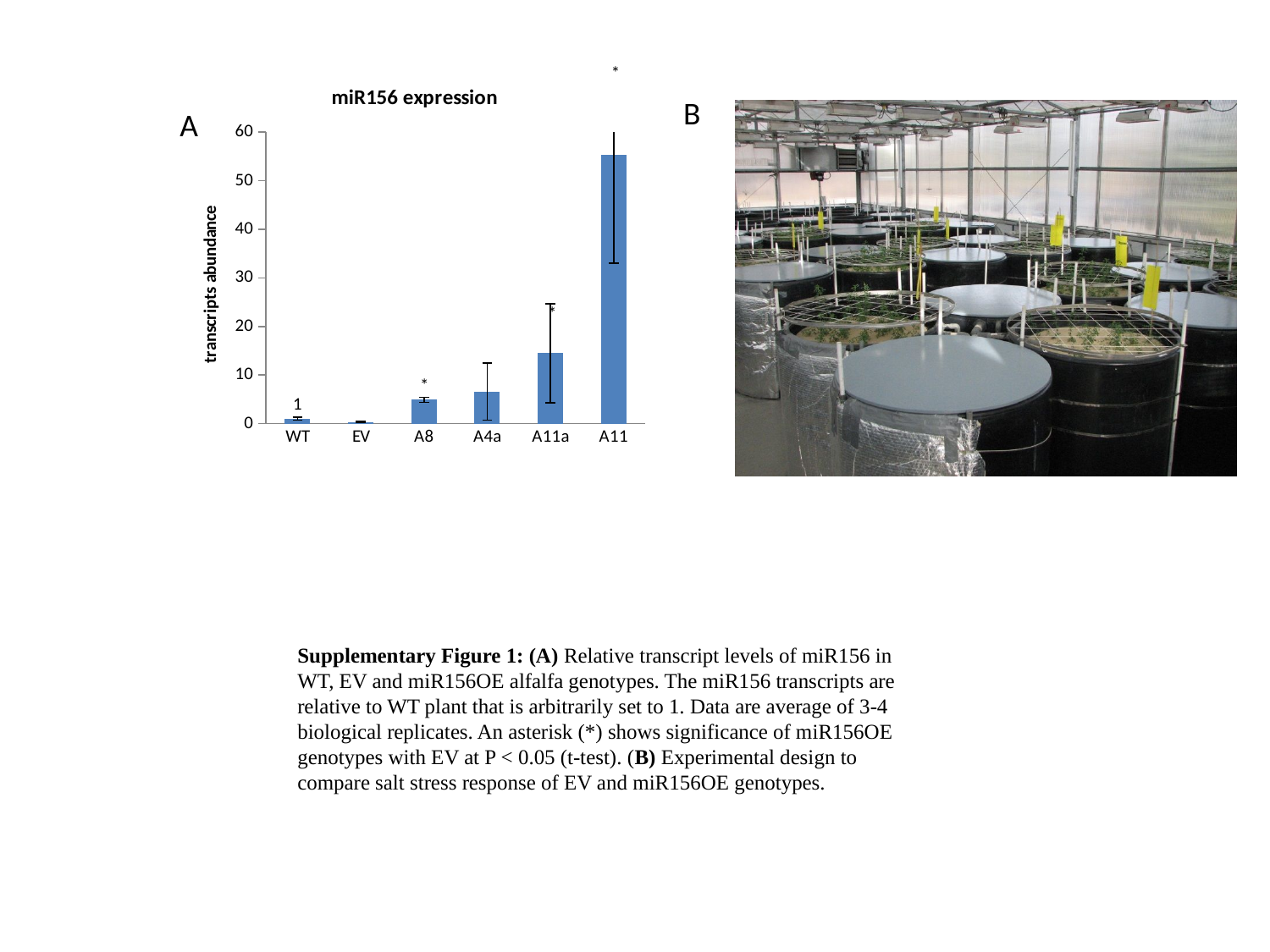

### Chart: miR156 expression
| Category | |
|---|---|
| WT | 1.0 |
| EV | 0.3 |
| A8 | 4.9 |
| A4a | 6.6 |
| A11a | 14.5 |
| A11 | 55.3 |B
A
Supplementary Figure 1: (A) Relative transcript levels of miR156 in WT, EV and miR156OE alfalfa genotypes. The miR156 transcripts are relative to WT plant that is arbitrarily set to 1. Data are average of 3-4 biological replicates. An asterisk (*) shows significance of miR156OE genotypes with EV at P < 0.05 (t-test). (B) Experimental design to compare salt stress response of EV and miR156OE genotypes.

## Slide 3
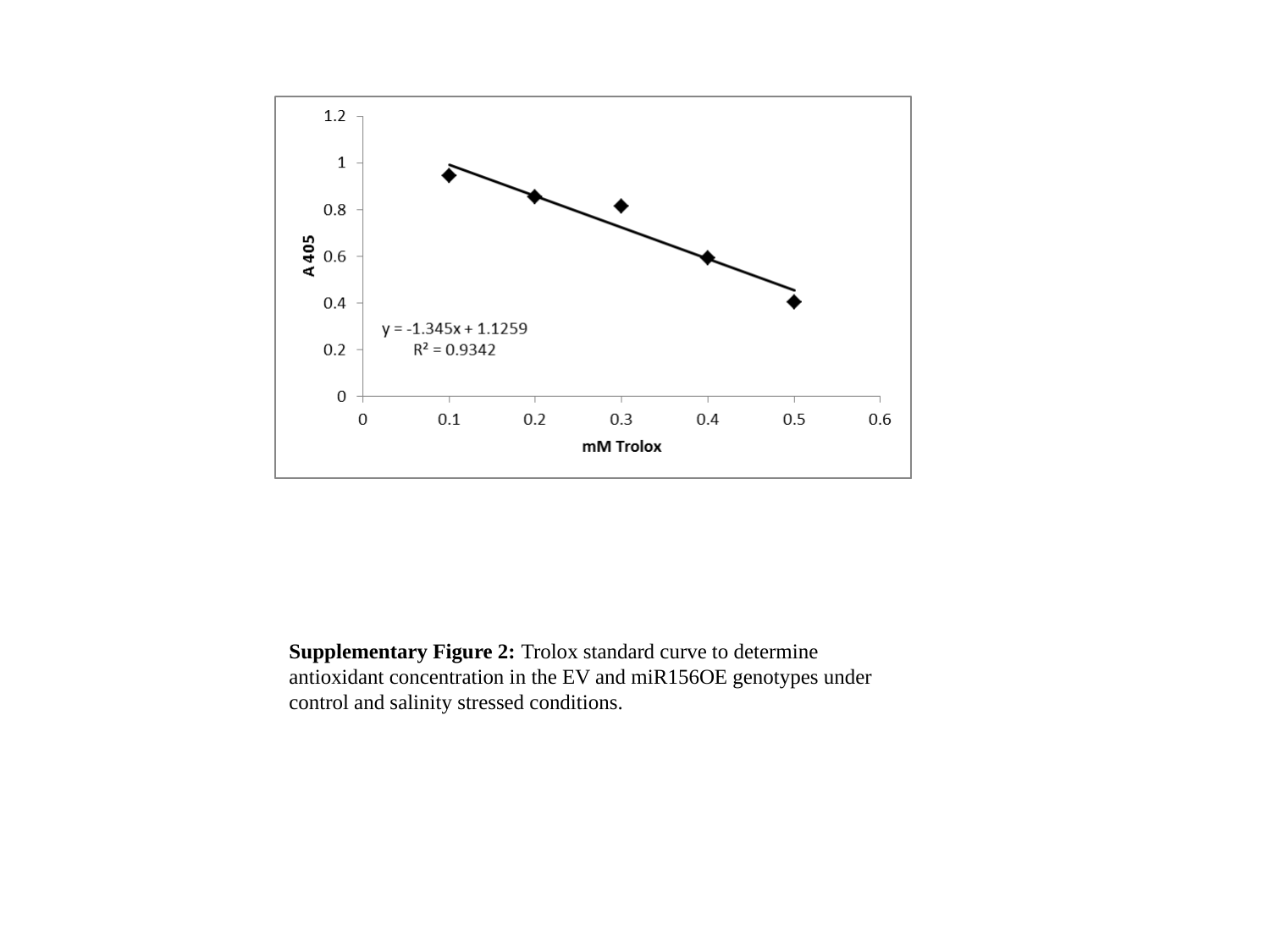

Supplementary Figure 2: Trolox standard curve to determine antioxidant concentration in the EV and miR156OE genotypes under control and salinity stressed conditions.

## Slide 4
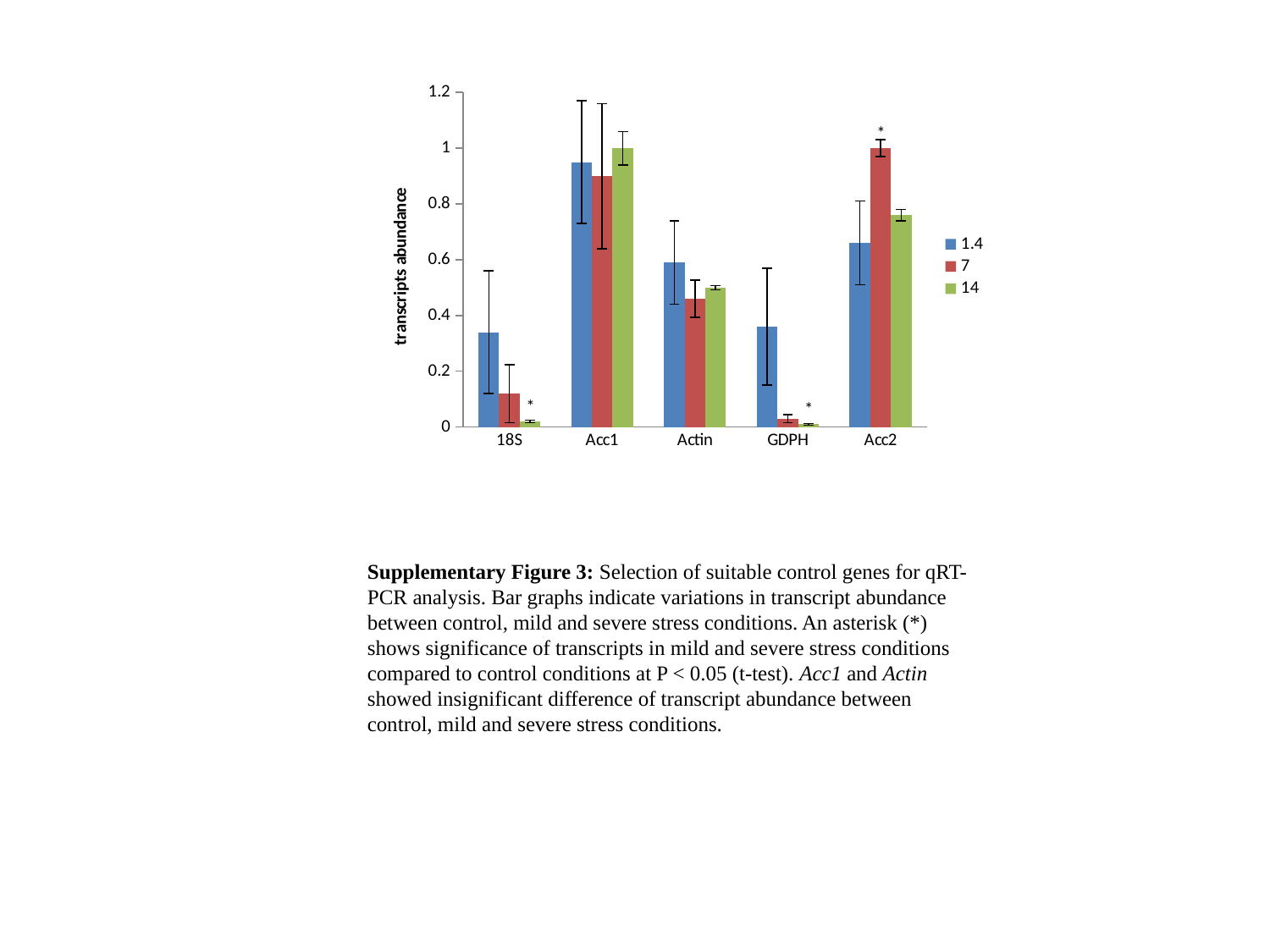

### Chart
| Category | 1.4 | 7 | 14 |
|---|---|---|---|
| 18S | 0.34 | 0.12 | 0.02 |
| Acc1 | 0.95 | 0.9 | 1.0 |
| Actin | 0.59 | 0.46 | 0.5 |
| GDPH | 0.36 | 0.03 | 0.01 |
| Acc2 | 0.66 | 1.0 | 0.76 |Supplementary Figure 3: Selection of suitable control genes for qRT-PCR analysis. Bar graphs indicate variations in transcript abundance between control, mild and severe stress conditions. An asterisk (*) shows significance of transcripts in mild and severe stress conditions compared to control conditions at P < 0.05 (t-test). Acc1 and Actin showed insignificant difference of transcript abundance between control, mild and severe stress conditions.

## Slide 5
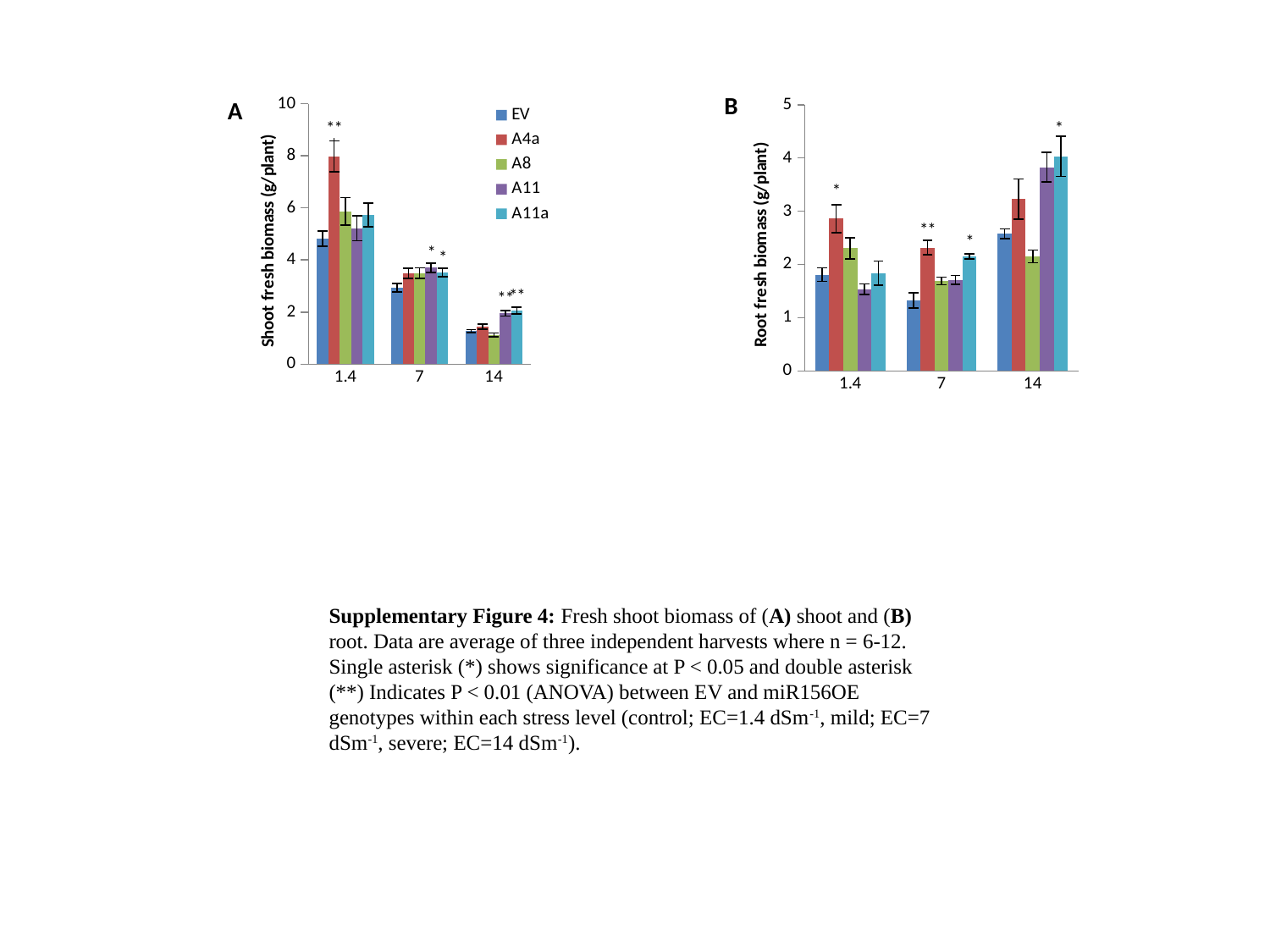

B
### Chart
| Category | EV | A4a | A8 | A11 | A11a |
|---|---|---|---|---|---|
| 1.4 | 4.818823529411765 | 7.984117647058823 | 5.870666666666668 | 5.21913043478261 | 5.7313636363636355 |
| 7 | 2.9339999999999997 | 3.4903448275862057 | 3.4996875 | 3.69939393939394 | 3.5206666666666653 |
| 14 | 1.2641176470588236 | 1.4429411764705886 | 1.116764705882353 | 1.953529411764706 | 2.0625806451612907 |
### Chart
| Category | EV | A4a | A8 | A11 | A11a |
|---|---|---|---|---|---|
| 1.4 | 1.807101476301476 | 2.8588520534388913 | 2.302533065750793 | 1.53461854912764 | 1.835773803609869 |
| 7 | 1.3197134090612352 | 2.317592793974649 | 1.6898328316938713 | 1.7103194178121075 | 2.149710975214392 |
| 14 | 2.5768281939519686 | 3.2287306083343075 | 2.152509881422925 | 3.827006306092683 | 4.030445722180816 |A
Supplementary Figure 4: Fresh shoot biomass of (A) shoot and (B) root. Data are average of three independent harvests where n = 6-12. Single asterisk (*) shows significance at P < 0.05 and double asterisk (**) Indicates P < 0.01 (ANOVA) between EV and miR156OE genotypes within each stress level (control; EC=1.4 dSm-1, mild; EC=7 dSm-1, severe; EC=14 dSm-1).

## Slide 6
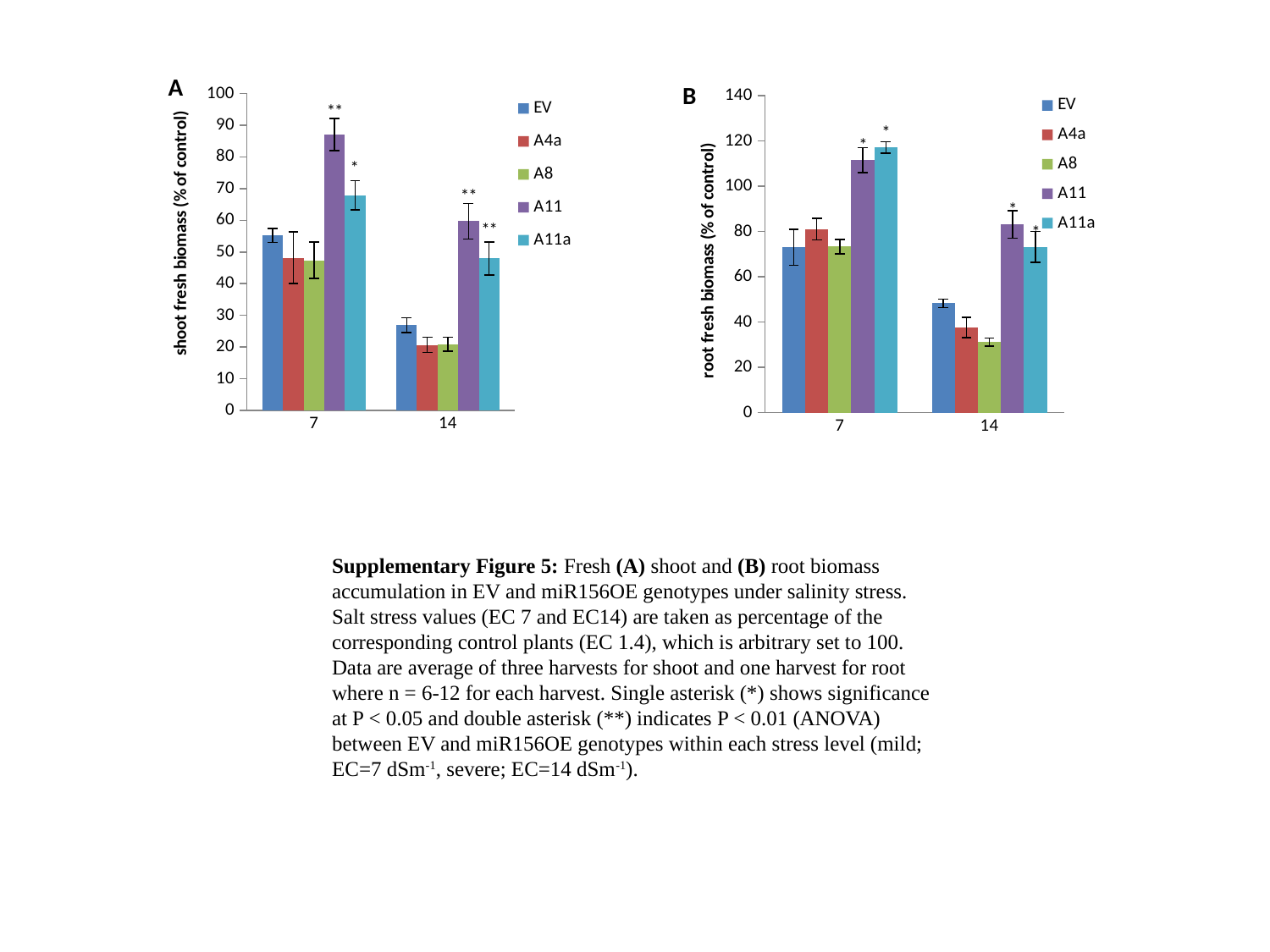

A
B
### Chart
| Category | EV | A4a | A8 | A11 | A11a |
|---|---|---|---|---|---|
| 7 | 55.2668194796929 | 48.17098255875036 | 47.41244749787292 | 87.05665806220964 | 67.94658077273608 |
| 14 | 26.91644292142592 | 20.681263195685823 | 20.94795755359711 | 59.716038359146395 | 47.98530502862399 |
### Chart
| Category | EV | A4a | A8 | A11 | A11a |
|---|---|---|---|---|---|
| 7 | 73.02929173419973 | 81.06725184280991 | 73.39016567576904 | 111.44915580385401 | 117.10108135257168 |
| 14 | 48.28784975137481 | 37.64600322065742 | 31.161476222869506 | 83.1261142661621 | 73.1834120713445 |Supplementary Figure 5: Fresh (A) shoot and (B) root biomass accumulation in EV and miR156OE genotypes under salinity stress. Salt stress values (EC 7 and EC14) are taken as percentage of the corresponding control plants (EC 1.4), which is arbitrary set to 100. Data are average of three harvests for shoot and one harvest for root where n = 6-12 for each harvest. Single asterisk (*) shows significance at P < 0.05 and double asterisk (**) indicates P < 0.01 (ANOVA) between EV and miR156OE genotypes within each stress level (mild; EC=7 dSm-1, severe; EC=14 dSm-1).

## Slide 7
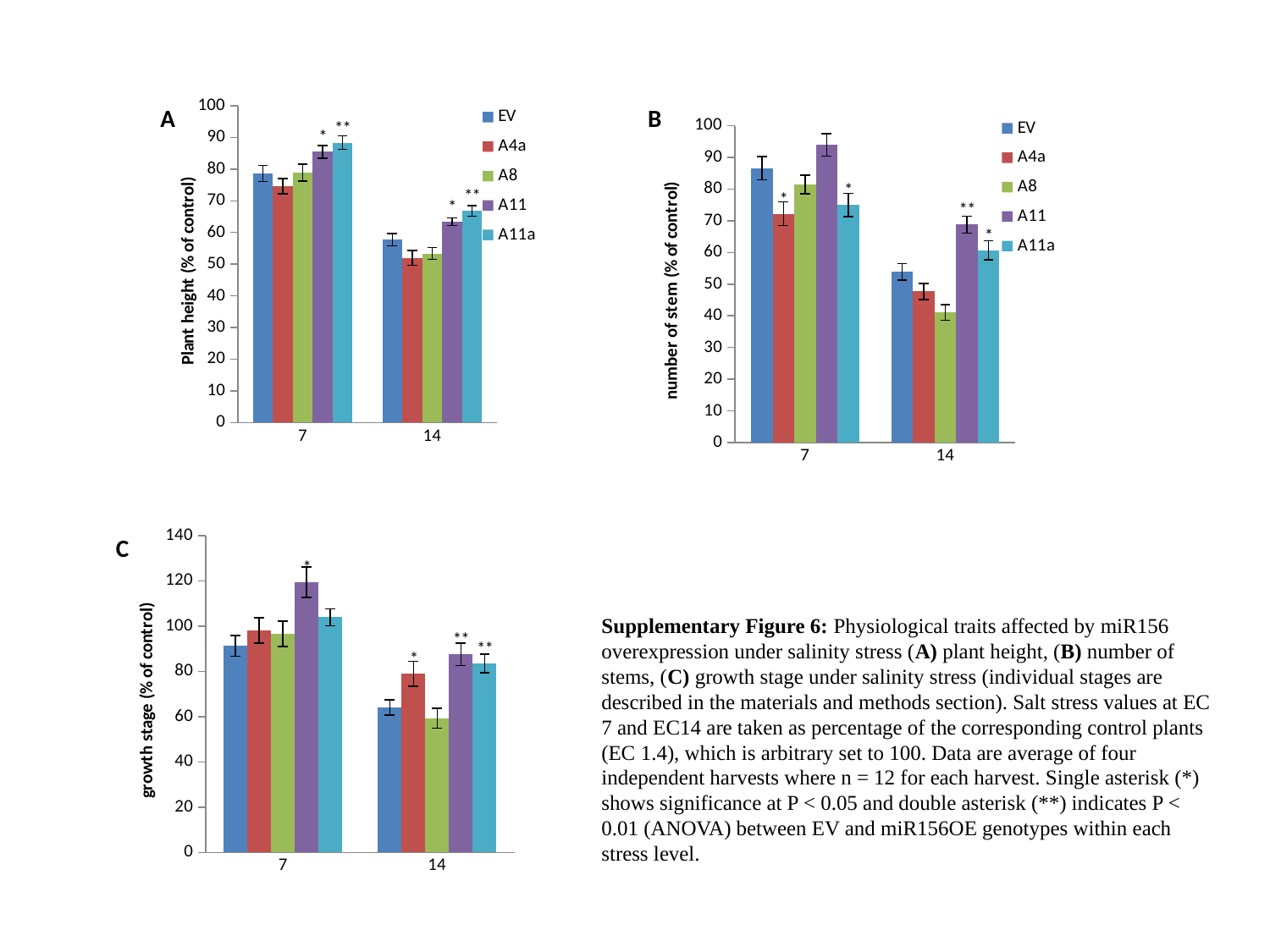

### Chart
| Category | EV | A4a | A8 | A11 | A11a |
|---|---|---|---|---|---|
| 7 | 78.64101746554718 | 74.58596435035484 | 78.92480929894658 | 85.4633894365287 | 88.33501328690558 |
| 14 | 57.760300421582166 | 52.00198787952737 | 53.37813294587719 | 63.45400542378712 | 66.80106295244211 |A
B
### Chart
| Category | EV | A4a | A8 | A11 | A11a |
|---|---|---|---|---|---|
| 7 | 86.57407407407406 | 72.23340040241426 | 81.45454545454571 | 93.90420899854841 | 74.93638676844783 |
| 14 | 53.93518518518518 | 47.68611670020106 | 41.090909090909186 | 68.79535558780826 | 60.68702290076337 |
### Chart
| Category | EV | A4a | A8 | A11 | A11a |
|---|---|---|---|---|---|
| 7 | 91.34199134199135 | 98.12206572769948 | 96.65071770334929 | 119.32773109243692 | 103.88349514563117 |
| 14 | 64.06926406926412 | 78.87323943661977 | 59.330143540669845 | 87.53799392097251 | 83.4951456310681 |C
Supplementary Figure 6: Physiological traits affected by miR156 overexpression under salinity stress (A) plant height, (B) number of stems, (C) growth stage under salinity stress (individual stages are described in the materials and methods section). Salt stress values at EC 7 and EC14 are taken as percentage of the corresponding control plants (EC 1.4), which is arbitrary set to 100. Data are average of four independent harvests where n = 12 for each harvest. Single asterisk (*) shows significance at P < 0.05 and double asterisk (**) indicates P < 0.01 (ANOVA) between EV and miR156OE genotypes within each stress level.

## Slide 8
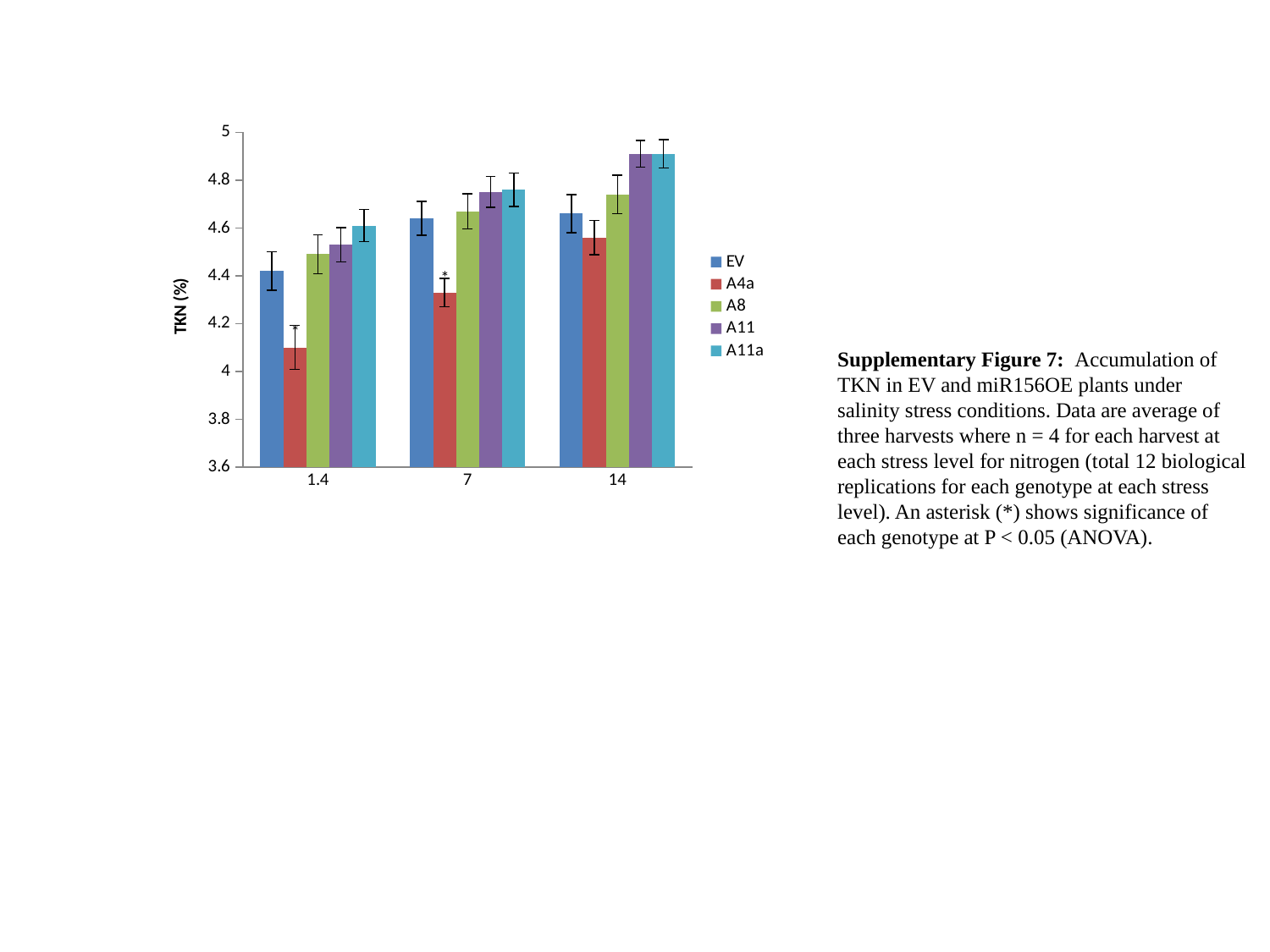

### Chart
| Category | EV | A4a | A8 | A11 | A11a |
|---|---|---|---|---|---|
| 1.4 | 4.42 | 4.1 | 4.49 | 4.53 | 4.61 |
| 7 | 4.64 | 4.33 | 4.67 | 4.75 | 4.76 |
| 14 | 4.66 | 4.56 | 4.74 | 4.91 | 4.91 |Supplementary Figure 7: Accumulation of TKN in EV and miR156OE plants under salinity stress conditions. Data are average of three harvests where n = 4 for each harvest at each stress level for nitrogen (total 12 biological replications for each genotype at each stress level). An asterisk (*) shows significance of each genotype at P < 0.05 (ANOVA).

## Slide 9
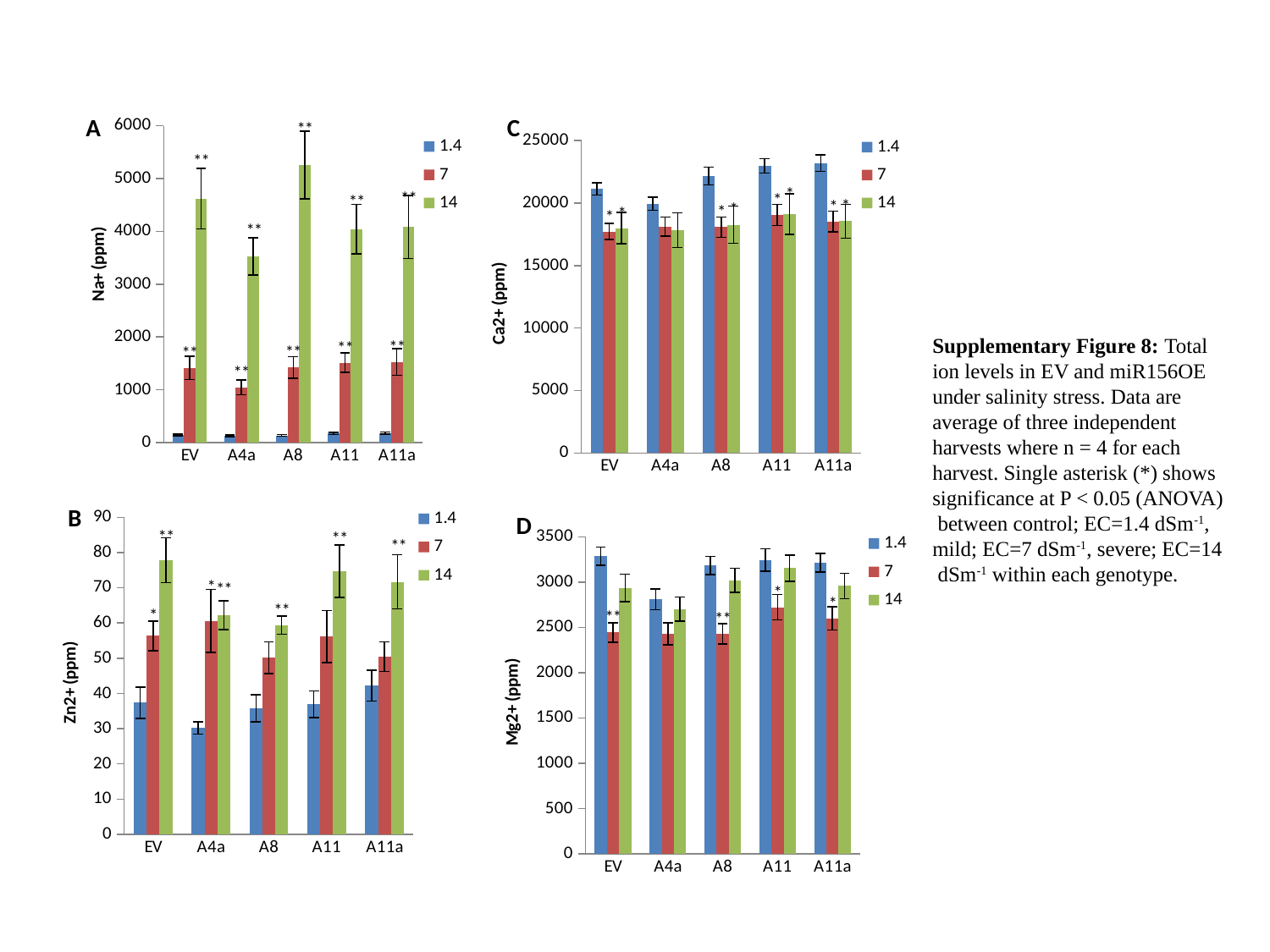

C
A
### Chart
| Category | 1.4 | 7 | 14 |
|---|---|---|---|
| EV | 143.0 | 1412.0 | 4620.0 |
| A4a | 128.3 | 1048.0 | 3527.0 |
| A8 | 135.6 | 1423.0 | 5253.0 |
| A11 | 171.6 | 1513.0 | 4042.0 |
| A11a | 179.8 | 1525.0 | 4081.0 |
### Chart
| Category | 1.4 | 7 | 14 |
|---|---|---|---|
| EV | 21132.0 | 17722.0 | 17989.0 |
| A4a | 19959.0 | 18125.0 | 17842.0 |
| A8 | 22156.0 | 18074.0 | 18268.0 |
| A11 | 22982.0 | 19059.0 | 19132.0 |
| A11a | 23192.0 | 18529.0 | 18548.0 |Supplementary Figure 8: Total
ion levels in EV and miR156OE
under salinity stress. Data are
average of three independent
harvests where n = 4 for each
harvest. Single asterisk (*) shows
significance at P < 0.05 (ANOVA)
 between control; EC=1.4 dSm-1,
mild; EC=7 dSm-1, severe; EC=14
 dSm-1 within each genotype.
B
### Chart
| Category | 1.4 | 7 | 14 |
|---|---|---|---|
| EV | 37.38 | 56.33 | 77.84 |
| A4a | 30.2 | 60.57 | 62.2 |
| A8 | 35.79 | 50.13 | 59.36 |
| A11 | 36.95 | 56.17 | 74.74 |
| A11a | 42.21 | 50.42 | 71.67 |D
### Chart
| Category | 1.4 | 7 | 14 |
|---|---|---|---|
| EV | 3286.0 | 2444.0 | 2935.0 |
| A4a | 2810.0 | 2429.0 | 2702.0 |
| A8 | 3184.0 | 2429.0 | 3021.0 |
| A11 | 3244.0 | 2723.0 | 3154.0 |
| A11a | 3212.0 | 2599.0 | 2957.0 |
